# Axonal Lysosomal Assays for Characterizing the Effects of LRRK2 G2019S

**DOI:** 10.3390/biology13010058

**Published:** 2024-01-20

**Authors:** Priyanka Bhatia, Marc Bickle, Amay A. Agrawal, Buster Truss, Aikaterina Nikolaidi, Kathrin Brockmann, Lydia Reinhardt, Stefanie Vogel, Eva M. Szegoe, Arun Pal, Andreas Hermann, Ivan Mikicic, Maximina Yun, Björn Falkenburger, Jared Sterneckert

**Affiliations:** 1 Center for Regenerative Therapies TU Dresden (CRTD), Technische Universität Dresden, 01307 Dresden, Germany; priyanka.bhatia@tu-dresden.de (P.B.);; 2 Roche Institute of Human Biology, 4070 Basel, Switzerland; 3 German Center for Neurodegenerative Diseases (DZNE), 72076 Tübingen, Germany; 4 Department of Neurodegenerative Diseases, Center of Neurology, Hertie Institute for Clinical Brain Research, University of Tübingen, 72076 Tübingen, Germany; 5 Department of Neurology, Technische Universität Dresden, 01307 Dresden, Germany; 6 Translational Neurodegeneration Section “Albrecht Kossel”, Department of Neurology, University Medical Center Rostock, University of Rostock, 18147 Rostock, Germany; 7 Center for Transdisciplinary Neurosciences Rostock (CTNR), University Medical Center Rostock, University of Rostock, 18147 Rostock, Germany; 8 Deutsches Zentrum für Neurodegenerative Erkrankungen (DZNE) Rostock/Greifswald, 18147 Rostock, Germany; 9 Max Planck Institute of Molecular Cellular Biology and Genetics, 01307 Dresden, Germany; 10 Physics of Life Excellence Cluster, 01307 Dresden, Germany; 11 Medical Faculty Carl Gustav Carus of TU Dresden, 01307 Dresden, Germany

**Keywords:** axonal trafficking, LRRK2, iPS cells, Parkinson’s disease, dying back

## Abstract

**Simple Summary:**

Axons are highly elongated extensions of neurons that transmit electrical impulses to their connecting targets and are integral for neuronal function. In neurodegenerative diseases such as Parkinson’s disease, axons degenerate early in the disease course, breaking essential connections, leading to the development of clinical phenotypes over time. Thus, developing a better understanding of axonal pathology is crucial. For this reason, we used patient-derived induced pluripotent stem cells to generate neurons which were then used to develop assays to characterize how neurodegenerative diseases such as Parkinson’s disease might affect axons. We show that LRRK2 G2019S, which is one of the most common known mutations causing Parkinson’s disease, subtly affects axonal function and the injury response. Our assays could be used in the future to better understand axonal degeneration and test potential therapeutics for their ability to protect axons against degeneration.

**Abstract:**

The degeneration of axon terminals before the soma, referred to as “dying back”, is a feature of Parkinson’s disease (PD). Axonal assays are needed to model early PD pathogenesis as well as identify protective therapeutics. We hypothesized that defects in axon lysosomal trafficking as well as injury repair might be important contributing factors to “dying back” pathology in PD. Since primary human PD neurons are inaccessible, we developed assays to quantify axonal trafficking and injury repair using induced pluripotent stem cell (iPSC)-derived neurons with LRRK2 G2019S, which is one of the most common known PD mutations, and isogenic controls. We observed a subtle axonal trafficking phenotype that was partially rescued by a LRRK2 inhibitor. Mutant LRRK2 neurons showed increased phosphorylated Rab10-positive lysosomes, and lysosomal membrane damage increased LRRK2-dependent Rab10 phosphorylation. Neurons with mutant LRRK2 showed a transient increase in lysosomes at axotomy injury sites. This was a pilot study that used two patient-derived lines to develop its methodology; we observed subtle phenotypes that might correlate with heterogeneity in LRRK2-PD patients. Further analysis using additional iPSC lines is needed. Therefore, our axonal lysosomal assays can potentially be used to characterize early PD pathogenesis and test possible therapeutics.

## 1. Introduction

Axons are essential for neuronal function, but their unique morphology makes them susceptible to degeneration. Since distal axons can be far away from the soma, their maintenance, by means of efficient intracellular transport, is particularly demanding. In addition, almost all central neurons are formed during development. Thus, axons must be maintained over an entire lifetime.

Parkinson’s disease (PD) is among the most prevalent neurodegenerative diseases, affecting an estimated 7 to 10 million people worldwide [1]. Although there are cases with onset as young as 40 years of age, PD primarily affects individuals aged 60 and older, and PD risk increases significantly with advancing age [2]. Clinically, PD develops as a movement disorder [3], but non-motor symptoms are experienced progressively during the course of the disease [4,5], leading to a consensus that PD shows broader involvement of various neuronal populations and brain regions [6,7]. A feature of PD is that neurons undergo “dying back” pathology in which axons degenerate months to years prior to the soma [8]. Imaging studies have not only revealed terminal loss in dopaminergic axons but have also indicated the affection of noradrenergic and serotonergic projections in PD patients [9,10,11]. Understanding how PD impacts axons is essential for understanding disease pathogenesis and developing protective treatments. Therefore, there is a critical need for in vitro models that can provide insights into axonal PD pathogenesis.

PD is associated with protein aggregates in both somas and axons [12], indicating that defects in protein quality control play an important and causal role in disease pathogenesis. Consistent with this idea, multiple genes encoding lysosomal enzymes, lysosomal membrane proteins, and proteins involved in lysosomal trafficking machinery are linked to PD, including *GBA*, *GALC*, *CTSB*, *CTSD*, *TMEM175*, *ATP6V0A1*, *LAMP3*, *ATP13A2*, *SLC17A5*, and *RABL1* [13,14,15]. Thus, lysosomes likely play an important role in PD, including in “dying back” pathogenesis. A model of human axonal lysosomes is needed to understand how their movement and localization may relate to PD pathology.

A particular challenge with PD is the clinical heterogeneity, including in age of onset and disease penetrance. For example, LRRK2 G2019S is one of the most common known PD mutations. However, PD with *LRRK2* mutations shows 67% penetrance [16] with a broad range in age at onset (34 to 73 years) [17], which is reminiscent of idiopathic cases. For this reason, *LRRK2*-PD is a good model for testing the sensitivity of axonal trafficking to detect early disease pathogenesis in neurons. LRRK2 is composed of the GTPase Roc-COR and kinase domains that are responsible for its catalytic activity [18] and house autosomal dominant PD-associated mutations, including G2019S, that increase its kinase activity [19]. The additional akryrin, leucine-rich repeat, and WD40 domains function as protein-protein and membrane interaction sites [20]. LRRK2 also possesses autophosphorylation activity that mediates its dimerization [21], which in turn has been shown to regulate its kinase activity [22], thereby playing a vital role in the protein’s physiological and pathological functioning. Studies have shown that LRRK2 mediates phosphorylation of Rab-GTPases, including Rab10, which regulates lysosomal dynamics [23,24,25,26]. LRRK2 G2019S has been associated with altered lysosome morphology, acidification, and reduced autophagic flux, leading to the accumulation of α-synuclein aggregates in mouse neurons [27]. The mutation has also found to increase LRRK2 recruitment and activity on damaged lysosomes in macrophages and astrocytes [25,26]. Since axons degenerate in early PD, this led us to hypothesize that lysosomal dysregulation could be an important contributing factor in axonal degeneration in *LRRK2*-PD. Thus, assays focusing on axonal lysosomes would be an important tool for studying early PD pathogenesis, including for LRRK2 cases as well as other disease subtypes.

Induced pluripotent stem cells (iPSCs) hold great promise for studying neurodegenerative diseases, including PD, because of their ability to generate patient-specific neurons, thereby facilitating the study of human neurons as opposed to murine neurons from animal models. Therefore, to better understand PD pathogenesis, we developed multiple assays for characterizing axonal lysosomes [28,29]. As proof of principle, we applied these assays to neurons differentiated from two previously generated LRRK2 G2019S-PD iPSC-derived intermediate neural progenitor cell (NPC) lines as well as isogenic controls [30,31].

## 2. Materials and Methods

### 2.1. Stem Cell Differentiation into Neurons

iPSCs and NPCs from the two patients as well as their isogenic gene-corrected controls were given to us by Prof. Hans Schöler’s team at the Max Planck Institute for Molecular Biomedicine (Münster, Germany), who derived them previously [30,31]. Neurons were differentiated from NPCs (between passage 12–17) by seeding 0.5 × 10^6^ cells (for Mut1, GC1) and 0.8 × 10^6^ cells (for Mut2, GC2) into a Matrigel Growth Factor Reduced (Corning) coated 6-well plate in patterning medium consisting of N2B27 medium supplemented with 200 µM ascorbic acid (Sigma Aldrich, St. Louis, MO, USA), 0.5 µM SAG (Cayman Chemical, Ann Arbor, MI, USA), 1 ng/mL GDNF (Peprotech, Waltham, MA, USA), and 1 ng/mL BDNF (Peprotech). After 7 days, neurons were matured using N2B27 medium supplemented with 200 µM ascorbic acid, 2 ng/mL GDNF, 2 ng/mL BDNF, 500 µM dbcAMP (Selleck Chemicals, Cologne, Germany), and 1 ng/mL TGFβ3 (Peprotech). A total of 5 ng/mL Activin A (BioLegend, San Diego, CA, USA) was added for the first two days. On the 14th day, neurons were reseeded as single cells in the desired cell culture system.

### 2.2. Treatment of Neurons with Compounds

All compound treatments were applied 3–4 weeks after reseeding of neurons in the final plate format. A 2 µM CZC-25146 (MedChemExpress, Monmouth Junction, NJ, USA) and 2 µM MLi-2 (MedChemExpress) treatment for lysosomal axonal trafficking experiments was applied for 48 h. For the LLOME (L-Leucyl-L-Leucine methyl ester)-mediated stress experiments, 1 µM LLOME (Sigma Aldrich) treatment was applied for 2 h. A 1 µM MLi-2 treatment was applied 90 min before LLOME treatment; hence, treatment was applied for 3.5 h in total. All treatments included the DMSO control that matched the maximum concentration and maximum treatment duration. Compounds were dissolved in maturation medium.

### 2.3. Seeding Neurons into Microfluidic Chambers (MFCs)

For compartmentalization, RD900 microfluidic devices (Xona Microfluidics, Temecula, CA, USA) were used. FluoroDish 35 mm glass bottom dishes (WPI, FD35-100) were coated with 30% Poly-L-Ornithine (PLO) (Sigma Aldrich) in DPBS (600 µL/dish) overnight at 37 °C. The coated FluoroDishes were washed 3 times with sterile water and, along with 70% ethanol-washed MFCs, they were exposed to UV light for 30 min, followed by air drying under sterile conditions. The MFCs were then assembled on the FluoroDishes by carefully pressing the device on the glass surface using a pair of forceps for firm adherence. The MFCs were perfused with 1:20 Laminin-521 (BioLamina, Sundbyberg, Sweden) in DPBS with 0.9 mM CaCl_2_ and 0.5 mM MgCl_2_ at 4 °C overnight for adherence and long-term culture of differentiated neurons. The next day, before seeding neurons, the laminin was removed from the MFCs and they were pre-incubated with N2B27 medium at 37 °C for 30 min.

For reseeding, neuronal cultures were gently dissociated with pre-warmed Accutase (Sigma Aldrich) at 37 °C and the cell suspension was passed through a sterile 70 µM cell strainer (Clear Line 141379C) to exclude any big clumps of cells. The coated MFCs were washed once with medium and 10 µL of cell suspension was injected directly into one well such that the cells occupied one of the two compartments, now referred to as the proximal compartment. Depending on survival and branching potential, to ensure optimal resolution of axons, the number of cells used for each line were as follows: Mut1 (0.25 × 10^6^ cells/MFC), GC1 (0.25 × 10^6^ cells/MFC), and Mut2 (0.2 × 10^6^ cells/MFC), GC2 (0.1 × 10^6^ cells/MFC). The MFCs were incubated for 30 min at 37 °C for initial attachment of the cells. Then, the wells on both sides were filled with maturation medium containing 5 μM ROCK inhibitor (Selleck Chemicals). Two days after seeding, axonal outgrowth was stimulated by establishing a neurotrophin concentration gradient while maintaining a difference in hydrostatic pressure between the two compartments. This was achieved by feeding the proximal compartment of the device that contains the neurons with 50 µL/well N2B27 supplemented with 200 µM ascorbic acid and 100 µM dbcAMP, while the distal compartment was supplied with maturation medium. This would enable directional growth of the long and fast-growing axons through the microchannels, while slow-growing dendrites would only penetrate the initial parts of the microchannels [32]. In the first medium change two days later, the maturation medium was supplemented with 5 µM DAPT (Cayman Chemical) to additionally boost maturation. Thereafter, the medium was replaced every two to three days depending on the medium consumption of the culture. After about a week, the first axons began spreading out at the distal site and the medium was changed to maturation medium in both compartments. After two weeks, axons were growing through majority of the microchannels and the MFCs were used for experiments.

### 2.4. Protein Extraction and Capillary Electrophoresis

In order to quantify the protein levels of Rab10 and phosphorylated Rab10 after LLOME and MLi-2 treatments, we used capillary electrophoresis. For sample preparation, after treatments, neurons were lyzed using RIPA buffer supplemented with protease inhibitor cocktail (Santa Cruz Biotechnology, Dallas, TX, USA) and PhosStop (Roche, Basel, Switzerland). Protein concentrations were determined using the Pierce BCA Protein Assay Kit (ThermoScientific, Waltham, MA, USA) and absorbance was measured on a Synergy Neo plate reader (BioTek, Winooski, VT, USA). Sample concentrations were calculated by interpolating detected values with those of the BSA standard curve. The neuronal protein lysates were analyzed using the 12-230 Separation Module of the Protein Simple WES capillary electrophoresis device. Quantification of the area under the curve of the specific peak at 30 kDa was performed using the software Compass for SW and relative levels of phosphorylated Rab10 were calculated by normalization with total Rab10 levels of the respective treatment condition. All reagents except primary antibodies were provided by the manufacturer (Bio-Techne, Minneapolis, MN, USA) and steps were performed according to the manufacturer’s instructions. Primary antibodies included: 1:750 rabbit anti-Rab10 (Abcam ab237703) and 1:10 rabbit anti-Rab10 phospho T73 (Abcam ab243293).

### 2.5. Axotomy

A total of 3–4 weeks after reseeding, axotomy was performed such that only the axons in the distal compartment were severed, without disturbing the cell bodies in the proximal compartment. Briefly, repeated vacuum aspiration of the axons in the distal compartment was performed by placing a tip connected to the laboratory vacuum close to the entrance of the distal compartment until the entire compartment was empty and the axons were cut effectively starting from the exit site of the microchannels. Both wells connecting the distal compartment were then filled with maturation medium and the MFCs were incubated at 37 °C. A total of 6 chambers per cell line were subjected to this procedure and each chamber was fixed at a particular time point post-axotomy such that axon extension and lysosome features could be measured across a time course. For each time point, lysosomes were labelled with 100 nM LysoTracker Deep Red (ThermoFisher Scientific), followed by 30 min incubation. Then, the neurons were fixed with equal volumes (as medium in wells) of 8% paraformaldehyde (Science Services, Munich, Germany) in DPBS for 15 min, followed by a DPBS wash. One MFC per cell line was fixed at 0.5, 17, 34, 51, 68, and 85 h post-axotomy. A no axotomy control was also fixed for each line, which consisted of a MFC from the same batch of differentiation that did not undergo axotomy.

### 2.6. Immunofluorescence

For immunostaining, neurons were fixed with 4% paraformaldehyde in DPBS for 20 min. Permeabilization and blocking of unspecific epitopes were performed simultaneously by applying 0.1% Triton X-100, 10% FBS, and 1% BSA in DPBS for 45 min. Primary antibodies were diluted in staining buffer consisting of 0.1% BSA in DPBS and applied to the cells overnight at 4 °C. The cells were washed thrice with staining buffer for 5 min. Secondary antibodies were diluted in staining buffer and were added for 1 h at room temperature. The cells were washed thrice with staining buffer for 5 min, and 0.01 mg/mL Hoechst 33342 (AAT Bioquest, Pleasanton, CA, USA) was added in the second wash to stain the nuclei. The cells were left in DPBS and stored in the dark at 4 °C until imaging. For immunostaining in MFCs, the buffers were first added to one well of the two interconnecting compartments, allowed to flow through the compartment, and then added to the second well. The following primary antibodies and dilutions were used: chicken anti-MAP2 (1:2000, Abcam ab92434), goat anti-Sox1 (1:100, Bio-Techne AF3369), goat anti-Sox2 (1:100, Santa Cruz Biotechnology sc-17320), mouse anti-Nestin (1:200, Bio-Techne MAB1259), mouse anti-TUBB3 (1:1000, BioLegend 801202), rabbit anti-PAX6 (1:100, BioLegend 901302), rabbit anti-RAB10 (phospho T73) (1:100, Abcam ab243293), and rat anti-LAMP1 (1:100, Abcam ab25245). Secondary antibodies included: AlexaFluor 488 donkey anti-rabbit IgG (A21206), AlexaFluor 488 goat anti-mouse IgG (A21202), AlexaFluor 488 goat anti-mouse IgG2a (A21131), AlexaFluor 555 goat anti-chicken IgY (A21437), AlexaFluor 555 goat anti-rat IgG (A21434), AlexaFluor 568 donkey anti-goat IgG (A11057), AlexaFluor 568 donkey anti-mouse IgG (A10037), and AlexaFluor 647 donkey anti-mouse IgG (A31571), all from ThermoFischerScientific and at a 1:1000 dilution.

### 2.7. Imaging and Quantification

NPCs and neurons fixed to determine the presence of cell type specific markers and MFCs immunostained with TUBB3 were imaged using an inverted Zeiss-Axio Observe.Z1 Apotome2 microscope. MFCs immunostained to determine the localization of Rab10 phospho T73 (phospho-Rab10) and LAMP1 as well as LysoTracker-labelled lysosomes after axotomy were imaged on an inverted Zeiss confocal laser scanning microscope (LSM980).

MFCs immunostained with TUBB3 for the quantification of outgrowth of axons after axotomy were imaged by acquiring tile images spanning the entire distal compartment, with 3 z-slices at an interval of 1 µm (since axons overlap each other in the z-direction). Orthogonal maximum projection of z-stack and stitching of tiles was performed using Zen 2.6 Pro software. Image analysis was performed using ImageJ. Briefly, pre-processing was performed to reduce noise by subtracting background with a rolling ball radius of 50 pixels. Gamma was adjusted to 0.7 and the image smoothened by applying a Gaussian filter with sigma 2. The images were then binarized by manual thresholding (keeping this value the same for all conditions of the experiment), particles smaller than 50 pixels were removed with the Particle Remover plugin, and the created mask was applied to the original image of the TUBB3 channel. Area and raw integrated density of the region occupied by TUBB3-positive axons were measured. Statistical analysis was performed using a 2-way ANOVA followed by Sidak’s multiple comparison test on GraphPad Prism (version 8.4.3).

Images for phospho-Rab10 and LAMP1 colocalization were captured at the entry (proximal) and exit (distal) sites of the microchannels. LysoTracker-labelled lysosomes after axotomy were imaged at the area in the distal compartment adjacent to the exit site of the microchannels. A z-stack with an interval of 0.5 µm was captured. At least 4 images per condition were acquired. Quantification of colocalization of phospho-Rab10 and LAMP1 was performed using the JaCoP plugin [33] in ImageJ. Briefly, the threshold of the two images was manually chosen (based on single channel controls) and calibration acquired from the metadata of the two images. Mander’s coefficient was computed by the plugin. Colocalization based on distance between the center of mass of objects detected after thresholding was also produced as an output. The proportion of the LAMP1-positive puncta colocalizing with phospho-Rab10 was calculated by dividing colocalizing centers of LAMP1 by total number of LAMP1 puncta, as computed by the plugin. The Mann-Whitney test was used for statistical analysis using GraphPad Prism. Image analysis of LysoTracker-labelled lysosomes was performed on CellProfiler. Briefly, first, TUBB3-positive axons were identified by pre-processing and thresholding using the Otsu method [34]. To ensure that connected tubular axons were identified, round particles with lower eccentricity and those with no neighbors were filtered, the mask generated was applied to the LysoTracker channel, lysosomes were detected by Otsu thresholding, and parameters like number, area, and intensity were obtained. For quantification, the number of lysosomes was normalized to the TUBB3-positive area. Statistical significance was determined by a 2-way ANOVA with Sidak’s multiple comparison test using GraphPad Prism.

### 2.8. Live Cell Imaging

To investigate the dynamics of lysosomes in axons of mutant and gene-corrected neurons, live cell imaging was performed as described by Naumann et al. [35] at specific regions of axons in the MFCs. A total of 3–4 weeks after the reseeding of neurons in MFCs, they were treated with the LRRK2 inhibitor compounds CZC-25146 and MLi-2 (along with DMSO control) and 30 min before the imaging session they were labelled with 100 nM LysoTracker Red DND-99 (ThermoFisher Scientific) added directly to the maturation medium and incubated at 37 °C. Without further washing, live cell imaging was performed using a Leica HC PL APO 100× 1.46 Oil objective on a Leica DMI6000 inverted fluorescent microscope housed in an incubator chamber (37 °C, 5% CO_2_). The excitation of LysoTracker Red DND-99 was determined using a 561 nm DPSS laser line and emission filter TRITC 605/65 nm. Fast dual color movies were recorded at 3.3 frames per second (fps) over 2 minutes (400 frames in total) with 115 ms exposure time. Readout positions chosen were just adjacent to the exit of microchannels (distal) and entry of the microchannels (proximal). The standardized set up covered two microchannels in parallel with a length of 117.53 µm at each readout position. About 10 movies were acquired focusing on different pairs of randomly selected microchannels at each readout position (distal and proximal) for each line, condition, and experiment. This resulted in a minimum of 30 movies in total for the batch analysis of one experiment. A total of 6–8 experimental replicates using neurons differentiated from a fresh passage of NPCs were performed.

### 2.9. Quantification of Axonal Trafficking Using Kymographs

Image analysis of trafficking of lysosomes was performed using the LysoTracker movies captured. The first few steps were performed using ImageJ (version 1.51n) [36]. The movies were first corrected by reducing the noise and subtracting background with a rolling ball radius of 50 pixels. Then, the dynamic movies were converted into a single image by generating a kymograph, which is a graphical representation of the movement of lysosomes in space along the x-axis and in time along the y-axis. This was undertaken by first using a segmented line (with width 31) to mark the region of interest that spanned the entire microchannel housing the axons (and lysosomes). Since each movie consisted of two microchannels, two regions of interest were marked. Kymographs were then generated using the KymoBuilder plugin. The kymographs were then analyzed in an automated fashion using KymoButler (version 1.1.4) [37], a deep learning Wolfram Mathematica (version 12.0.0.0)-based software. This software traces lines in the kymographs, taking into consideration certain user-defined parameters, and extracts quantitative information about particle movement. The BiKymograph function was used with a detection threshold of 0.10, minimum particle size of 10 pixels, and minimum frame number of 15 frames. Multiple combinations of these thresholds were tested, and one was selected based on its ability to faithfully trace continuous tracks in low-signal or sparsely or densely populated kymographs. For each kymograph analyzed, the output of the function consisted of a colored overlay with a randomly assigned color tracing each detected track, a CSV file with all track coordinates, and a summary file with quantities such as average speed, track duration, track direction, etc. The results from all conditions of one experimental replicate were assembled in KNIME (version 4.2.3) [38]. Track absolute displacement was calculated from the first and last track coordinates. Using KNIME version 4.5.0, with the required rtools support, the results from all experimental replicates were assembled. The tracks were divided into distal and proximal axonal regions and categorized as retrograde, anterograde, or stationary based on the track direction output from KymoButler. The tracks were then additionally filtered to include only long-range transporting lysosomes with track absolute displacement more than 4.5 µm. Frequency distribution graphs of speed and displacement were generated from the filtered population using R Plot nodes and normality ascertained by inspection of the histograms. After appropriate transformations, a two-way ANOVA with Tukey’s multiple comparison test was performed using the R snippet node comparing features such as average speed and displacement between conditions. Box-plots were generated using R Plot nodes, with batch results merged from all independent experimental replicates.

### 2.10. Quantification of Axonal Trafficking Using an Object-Based Method

As an additional form of analysis of axonal trafficking, individual lysosomes were detected within each image frame of the captured movies using CellProfiler (version 4.2.1) [39]. Briefly, speckles in each image were enhanced and lysosomes were segmented according to Otsu’s method. Each lysosome was then tracked across frames using the TrackObjects module based on the lysosomes appearing at a maximum distance of 4 pixels in subsequent frames, with a minimum lifetime of 12 frames. Successfully tracked lysosomes were labelled on the original image with each lysosome appearing in a unique color. Quantitative measurements such as distance travelled, integrated displacement, final age, and x & y trajectory of each tracked lysosome along with the metadata were saved as a text file. Since all the experiments along with their experimental replicates generated a large number of movies with terabytes worth of data, a high-performance computing cluster was used to perform the CellProfiler analysis. A Snakemake [40] workflow with each step to be performed was created for reproducible analysis and the job submitted to the cluster. Once the quantitative measurements from each experiment were saved, the results were assembled in KNIME. Following the filtering out of lysosomes with a displacement less than 4.5 µm, the speed of each lysosome was calculated, and a direction assigned as anterograde or retrograde based on its trajectory along the x-axis. As described in the previous section, frequency distribution graphs were generated and normality ascertained. After appropriate transformations, a two-way ANOVA with Tukey’s multiple comparison test was performed comparing features such as average speed and displacement between conditions. Box-plots were generated with batch results merged from all independent experimental replicates.

### 2.11. Clinical and Biomarker Data Collection

The two patients reported in this study were patients of the outpatient clinic for Parkinson’s disease at the University of Tübingen. In total, longitudinal cerebrospinal fluid (CSF) data from at least 2 time points were available. At each time point, the subjects were examined by a movement disorder specialist. Diagnosis of PD was defined according to UK Brain Bank Society Criteria [41] and the severity of symptoms was assessed using part III of the Movement Disorder Society Unified Parkinson’s Disease Rating Scale (UPDRS-III) [42]. Disease stage was categorized by using the modified Hoehn and Yahr Scale (H&Y) [43]. Cognitive function was tested using the Montreal Cognitive Assessment (MoCA) [44]. Levodopa-equivalent daily dosage (LEDD) was calculated according to the guidelines of the German Society for Neurology (http://www.dgn.org/ accessed on 1 October 2008).

CSF collection and determination of routine diagnostic parameters were performed according to standardized protocols [45]. In brief, a spinal tap was performed between 9.00 am and 1.00 pm after a fasting period of at least 6 h. The subjects were in a sitting position and the needle was tapped in the L3–4 or L4–5 interspace. Samples were taken directly from the bedside and centrifuged within 30 min after collection and frozen at −80 °C within 60 min after collection. Only samples of subjects with normal routine CSF diagnostics (white blood cell count < 4 × 10^6^/L, IgG index < 0.6) were included. CSF levels of Aβ1–42, total Tau, and phosphorylation of Tau at T181 were determined using commercially available ELISA kits (Innogenetics NV, Ghent, Belgium). Genotyping of *MAPT* H1/H2 haplotype, *SNCA* SNP rs356220, and *APOE* haplotype was performed using a Neurochip array [46].

## 3. Results

### 3.1. Establishing an Axonal Lysosomal Trafficking Assay

PD pathogenesis follows a “dying back” pathology with axons degenerating very early in the disease course. Additionally, there is a prominent involvement of lysosomal proteins in LRRK2-PD pathogenesis. We speculated that axonal trafficking of lysosomes could be a sensitive assay for detecting early PD pathogenesis, which could be particularly useful for disease cases such as LRRK2 G2019S that are late onset and linked to partially penetrant genetic mutations. Previously, we generated two sets of isogenic patient-derived iPSCs [30], which were differentiated into NPCs [31]. Immunostaining showed that all four NPC lines expressed the neural progenitor markers Nestin, PAX6, SOX1, and SOX2 (Figure S1A,B). The neuronal identity of the cells differentiated from the NPCs was confirmed by observing the presence of the pan-neuronal markers MAP2 and TUBB3 using immunostaining (Figure S1C). Additionally, phospho-proteomics data showed expression of MAP2, TUBB3, NRCAM, SYN1, STMN2, SNAP25, ANK3, CHD7, GABARAPL2, GAD1, and TH, indicating a mixed neuronal culture containing glutamatergic, GABAergic, and dopaminergic neurons.

**Figure 1:**
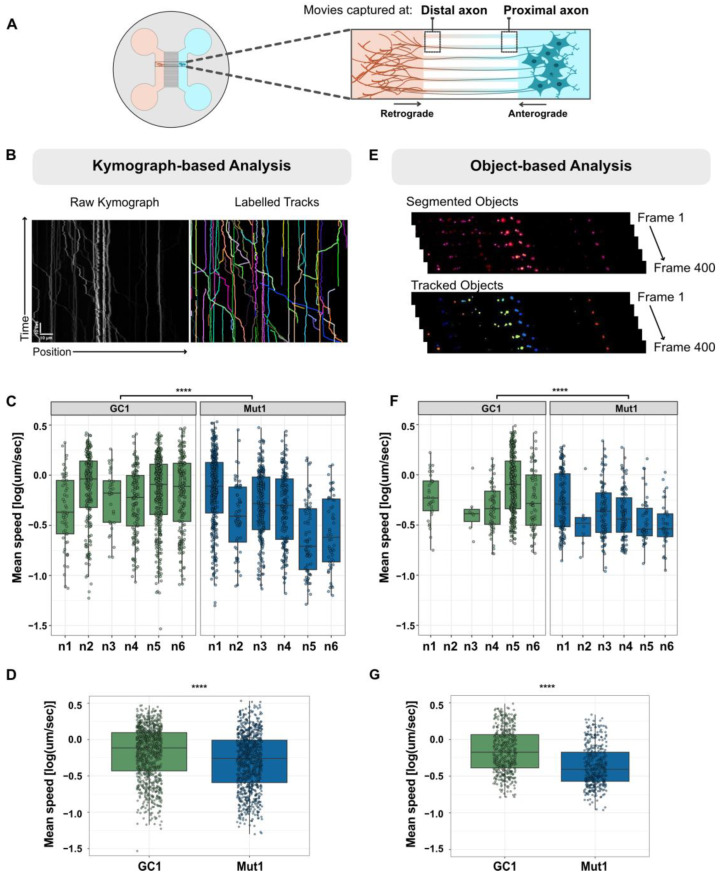
Axonal trafficking experimental setup and analysis methods. (**A**) Schematic representation of MFC used to capture retrograde and anterograde lysosomes moving in distal and proximal axons. (**B**) Representative kymograph and labelled tracks of lysosomes generated using kymograph-based analysis. Scale bar horizontal = 10 μm, vertical = 10 s. (**C**) Box-plot representing the variability of the mean speed of lysosomes between experimental replicates (n1–n6) of GC1 and Mut1 quantified from kymograph-based analysis (each point in the box represents an individual lysosome). (**D**) Pooled values of the mean speed of lysosomes from all six experimental replicates of GC1 and Mut1 were quantified using kymograph-based analysis. (**E**) Representation of object-based analysis, where individual objects were segmented and tracked from frames 1 to 400. (**F**) Box-plot representing the variability of the mean speed of lysosomes between experimental replicates (n1–n6) of GC1 and Mut1 quantified from object-based analysis. (**G**) Pooled values of the mean speed of lysosomes from all six experimental replicates of GC1 and Mut1 were quantified using object-based analysis. Statistics were calculated using a 2-way ANOVA with the post-hoc Tukey’s test. **** corresponds to *p*-adj ≤ 0.0001.

Developing axonal assays requires a tool to isolate axons from the rest of the neuron. To achieve this, microfluidic chambers (MFCs) were used to spatially separate the axons from the soma and dendrites [32,35,47]. These MFCs consist of two different compartments connected by a 900 µm microchannel barrier (Figure 1A and Figure S2). The differentiated neurons were seeded into one side of the MFCs, referred to as the proximal compartment. Application of a higher neurotrophin concentration on the opposite side created a gradient that stimulated the outgrowth of axons, marked by TUBB3, through the microchannels into the distal compartment. Since the microchannels were too narrow, the soma did not migrate to the distal compartment. Immunostaining confirmed that short and slow-growing dendrites, marked by MAP2, remained largely in the proximal compartment, and could penetrate only the initial parts of the microchannels (Figure S2), consistent with previous reports [48].

We aimed at establishing an axonal lysosomal trafficking assay to characterize possible PD-associated defects. We chose to apply this assay to neurons with LRRK2 G2019S, which is the most common known LRRK2-PD mutation. To assess axonal trafficking, lysosomes were labelled with LysoTracker Red DND 99 two weeks after seeding neurons in the MFCs. LysoTracker is a cell-permeable fluorescent dye that is selective to acidic organelles. Live imaging of lysosomes was performed by capturing movies in proximal and distal axons (position) for 2 min (Figure 1A). About 10 videos were captured for each condition and position (technical replicates), and at least six experimental replicates were performed with fresh batches of differentiated neurons.

The compiled movies from each live cell imaging experiment resulted in very large data volumes. In order to significantly reduce the file sizes of movies and enable easy visualization of moving as well as stationary lysosomes, we first generated kymographs that are graphical representations of the position of objects across the x-axis and over time across the y-axis (Figure 1B). Then, we used a deep learning algorithm, KymoButler [37], to automate tracing, circumventing the laborious, bias-prone manual annotation method. Among several parameters, as an output, the software provided quantification of average speed and displacement for individual lysosomes. The data were then mined by segregating lysosomes according to directionality (anterograde, retrograde) and position (proximal, distal) and focusing on long-range transporting lysosomes with a net displacement exceeding 4.5 µm.

Unfortunately, there was considerable variation between experimental replicates (Figure 1C), possibly due to variations in differentiation, culture conditions, and microenvironment. Thus, we sought to develop a methodology that could identify differences arising due to the genotype (e.g., LRRK2 G2019S versus gene-corrected) while taking into consideration the experimental variation. First, we normalized the data distribution by applying a log transformation. Then, we employed the 2-way ANOVA to separately examine the influence of the genotype and experimental replicates on the speed and displacement of lysosomes. This was followed by a post-hoc Tukey’s test for multiple comparisons to identify which groups within the genotype were different from each other and report the differences in their means. In this manner, the significance of LRRK2 G2019S-associated differences could be calculated while taking into consideration the variation coming from each replicate. To simplify the representation, we pooled together all the data points collected from all replicates and reported differences with the p-value calculated as described above (Figure 1D).

The large number of individual axonal lysosomes being quantified meant that even very small changes were often statistically significant (*p* < 0.05). This implied that even minor alterations in the analysis pipeline had the potential for amplifying or even creating spurious results. Thus, it was important to ensure that any significant differences observed in axonal lysosomal trafficking were independent of the algorithm being used for quantification. Additionally, we sought to complement the black box approach of KymoButler with an independent algorithm. For these reasons, individual lysosomes were segmented within each frame of the movie using CellProfiler [39], and each segmented lysosome was tracked across frames (Figure 1E). As with KymoButler, there was considerable variation in the parameters within and between experiments analyzed using the object-based method (Figure 1F). We also observed that the total number of lysosomes tracked after filtering for a minimum displacement of 4.5 µm was lower than with the kymograph method, as indicated by fewer points in the box-plots of Figure 1F vs. Figure 1C. One possible explanation for this is that CellProfiler is unable to accurately follow objects coming in and out of focus, labelling them as new tracks each time they appear. This leads to shorter tracks that fall below the minimum 4.5 µm displacement.

### 3.2. The Axonal Trafficking Assay Detects LRRK2 G2019S-Associated Changes in Lysosome Movement

Once the axonal trafficking assay was established, we sought to further characterize lysosomal trafficking in *LRRK2*-PD neurons derived from the two patients. To provide a detailed assessment of the effects of LRRK2 G2019S on axonal lysosomal trafficking, we used neurons derived from both patients (Mut1 and Mut2) along with their respective isogenic gene-corrected controls (GC1 and GC2). As expected [49], there were more lysosomes undergoing retrograde transport to the soma than anterograde transport towards the axons (Figure S3). There was no significant difference between Mut1 and GC1 or between Mut2 and GC2 in the proportion of lysosomes moving in each direction (Figure S3).

**Figure 2:**
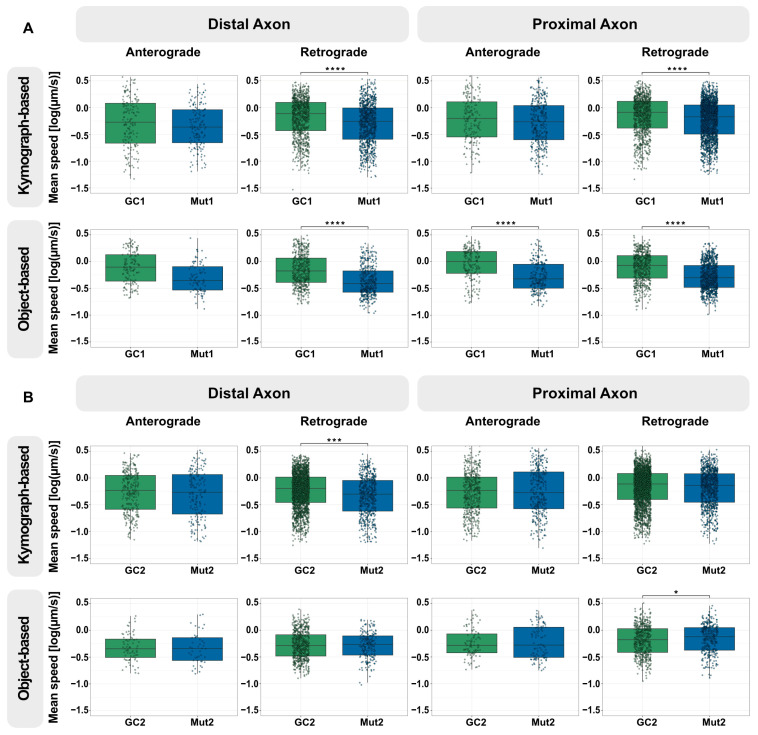
LRRK2 G2019S reduces the speed of retrograde lysosomal trafficking in neurons from one NPC line with mutant LRRK2 but not as much in those from the second. (**A**) Quantification of the mean speed of anterograde and retrograde lysosomes in the distal and proximal axons of GC1 and Mut1 neurons using kymograph-based analysis (top) and object-based analysis (bottom). (**B**) Quantification of the mean speed of anterograde and retrograde lysosomes in the distal and proximal axons of GC2 and Mut2 neurons using kymograph-based analysis (top) and object-based analysis (bottom). For all graphs: Pooled individual measurements from N = at least six independent experimental replicates. Statistics were calculated using a 2-way ANOVA with the post-hoc Tukey’s test to compare GC and Mut, considering any variation between the experimental replicates. **** corresponds to *p*-adj ≤ 0.0001, *** to *p*-adj ≤ 0.001, and * to *p*-adj ≤ 0.05. Comparisons without a marked * did not show any significant differences. The exact mean differences and p-values can be found in Table S1. Graphical representations of individual experimental replicates can be found in Figures S4 and S5.

Next, we compared the distribution of speed of axonal lysosomal transport in Mut1 vs. GC1 neurons. For efficient maintenance of axons along their considerable length, axonal lysosomes are required to undergo long-range transport. Thus, we focused solely on lysosomes with a net displacement of at least 4.5 µm. Using kymograph-based analysis, we observed no significant differences in the anterograde speed of lysosomes in both distal and proximal axons, which could be due to the relatively low number of lysosomes undergoing anterograde transport (Figure 2A). The speed of Mut1 was significantly reduced compared with GC1 lysosomes undergoing retrograde transport in the distal axon. This difference was also present in the proximal axon, but the magnitude of the effect was smaller (Figure 2A, upper panel). Interestingly, the object-based algorithm not only detected a reduction in retrograde transport speed in Mut1 compared with GC1 neurons, but also a reduction in anterograde transport speed in the proximal axon (Figure 2A, lower panel). Overall, these results indicate that our axonal trafficking assay detects LRRK2 G2019S-associated changes in the movement of axonal lysosomes from this set of isogenic cell lines, with a more consistent effect on retrograde movement.

Following this result, we assessed if LRRK2 G2019S was associated with changes in lysosomal trafficking in the second group of isogenic differentiated neurons (Mut2 vs. GC2). Investigation of the kymographs revealed a similar reduction in the speed of mutant lysosomes towards the retrograde direction in the distal axon (Figure 2B, upper panel). In the proximal axon, even though the speed of lysosomes in either direction trended lower in the mutant, this was not significantly different. The object-based pipeline found no significant difference in the speed of Mut2 and GC2 lysosomes undergoing transport in the distal axon (Figure 2B, lower panel). We observed a minor increase in the speed of Mut2 proximal lysosomes in the retrograde direction compared with GC2 (Figure 2B, lower panel). A point to note is that the retrograde movement in the distal axons was identified to be significantly reduced in three of the four analyses (Mut1, kymograph-based; Mut1, object-based; and Mut2, kymograph-based) compared to in the proximal axons, indicating that the effect of the mutation might be slightly more consistent in the distal axons than the proximal regions (Table S1).

To further evaluate the movement of lysosomes, we compared the final displacement of the lysosomes from their initial position in both distal and proximal axons as a second parameter. Using kymograph-based analysis, we observed no significant differences in the anterograde displacement of lysosomes in both distal and proximal axons between Mut1 and GC1. Interestingly, there was a significant reduction in the displacement of Mut1 lysosomes moving in the retrograde direction in both regions (Figure S6, upper panel). This effect was detected confidently only in the distal axons using object-based analysis (Figure S6, lower panel). Taken together, both speed and displacement of lysosomes from the axons to the soma were reduced in the presence of LRRK2 G2019S in this isogenic pair. On comparing the displacement of lysosomes in the second isogenic pair, we observed that the mutant lysosomes moved further in both directions and regions of the axons, according to the kymograph-based analysis (Figure S7, upper panel). However, this was not found to be significantly different according to the object-based algorithm (Figure S7, lower panel), indicating no true effect.

Taken together, these results demonstrate that the axonal trafficking assay has the potential to detect small perturbations in the movement of lysosomes in neurons. Additionally, the effect of LRRK2 G2019S on the speed of lysosomes appears to be more consistent than that on their displacement. Considering that neurons affected in PD have extremely long and arborized axons, even small changes in axonal trafficking would add up to a huge difference in the overall movement of lysosomes through the dense network of axons. Since these cells are metabolically very demanding, an inefficiency in the transport of lysosomes could potentially lead to the buildup of toxic material and thus be an important contributing factor to dying back of axons in PD over time. Interestingly, the differences in axonal transport appear to be cell line specific. PD patients manifest considerable variation in onset and disease progression, and it is tempting to speculate that the axonal trafficking assay might be detecting patient-specific variation. However, more experiments would be needed to test that particular hypothesis. Nevertheless, this assay might be a useful tool for better understanding the heterogeneity within the axons of LRRK2 G2019S carriers.

### 3.3. Axonal Trafficking Assay Detects Partial Rescue by a Small-Molecule LRRK2 Inhibitor

Novel treatments are urgently needed to protect neurons from neurodegenerative diseases, including PD, and LRRK2 has emerged as a promising drug target. Since G2019S increases LRRK2 kinase activity, several potent and selective small molecules have been developed to inhibit LRRK2 kinase activity, thereby reducing disease pathogenesis. We selected compounds belonging to two different generations of LRRK2 kinase inhibitors—CZC-25146 and MLi-2. Both compounds have the same mode of action, i.e., they compete with ATP to bind to the kinase in the active site. CZC-25146 is a potent and selective small molecule with an IC_50_ of ~7 nM [50], however it does not cross the blood-brain barrier. It was selected because unpublished data from our lab showed promising results with its use. MLi-2 was chosen as the second inhibitor as it has an especially high potency with an IC_50_ of 0.7 nM [51], can cross the blood-brain barrier, and is closely related to a compound that has progressed to clinical trials.

**Figure 3:**
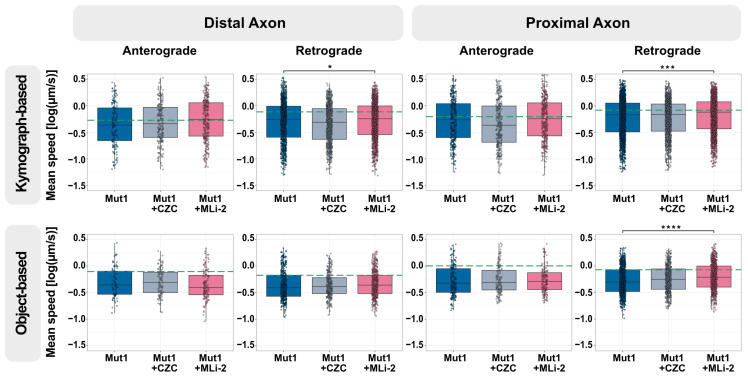
The LRRK2 kinase inhibitor, MLi-2, has a small effect on retrograde lysosome trafficking in the distal and proximal axon. Quantification of the mean speed of anterograde and retrograde lysosomes in the distal and proximal axons of Mut1 neurons without and with 2 µM LRRK2 kinase inhibitors CZC-25146 or MLi-2 for 48 h using kymograph-based analysis (top) and object-based analysis (bottom). The green dashed line marks the median lysosomal speed of GC1 in the specific direction and position. For all graphs: Pooled individual measurements from N = at least six independent experimental replicates. Statistics were calculated using a 2-way ANOVA with the post-hoc Tukey’s test to compute the effect of compound treatment on comparison with no treatment, taking into consideration any variation between the experimental replicates. **** corresponds to *p*-adj ≤ 0.0001, *** to *p*-adj ≤ 0.001, and * to *p*-adj ≤ 0.05. Comparisons without a marked * were not significantly different than Mut1. The exact mean differences and *p*-values can be found in Table S1.

Since axonal lysosomal transport was disrupted more in Mut1 neurons compared with in Mut2 neurons, we first tested if LRRK2 inhibitors would rescue this defect in this mutant line. Two weeks after seeding in the microfluidic chambers, neurons were treated with 2 µM CZC-25146 or 2 µM MLi-2 for 48 h followed by live cell imaging. Using kymograph-based analysis of the captured movies, we observed no effect with either compound on the speed of axonal lysosomal anterograde transport (Figure 3). In contrast, we found that MLi-2, but not CZC-25146, increased the speed of both distal and proximal axonal lysosomal retrograde transport compared to controls (Figure 3, upper panel). However, this increase was detected confidently using the object-based algorithm only in the proximal axons (Figure 3, lower panel).

Next, we assessed the displacement of lysosomes in the inhibitor-treated Mut1 axons. Similar to the speed, no change was observed with either compound in anterograde movement. Using kymograph-based analysis, we observed an increase in the displacement of proximal retrograde lysosomes on treatment with MLi-2 (Figure S8, upper panel); however, this was not detected confidently using the object-based algorithm (Figure S8, lower panel).

Since MLi-2 was associated with improved retrograde axonal transport of lysosomes, we asked if the effect size was sufficient to rescue the mutation-associated phenotype. However, it is clear that MLi-2 had a relatively small effect and did not entirely rescue the trafficking defect observed in Mut1 neurons (green dashed line, Figure 3).

Next, we assessed the effects of LRRK2 inhibitors on Mut2 neurons. However, it is important to note that we did not observe any reduction in the speed of lysosomes from Mut2 in comparison to its isogenic control GC2. Nevertheless, this experiment is important in order to have a complete picture of the effect of the LRRK2 inhibitors on lysosomal trafficking. As expected, we did not find any significant differences in treatment that could be identified by both forms of analyses (Figure S9).

None of the inhibitor treatment conditions were sufficient to completely rescue the distal trafficking defect in the Mut1 axons. One reason could be that *LRRK2*-PD phenotypes are modified by polymorphisms at other loci (not just within *LRRK2*) as well as environmental factors, which are not targeted by LRRK2-specific inhibitors. It could also be important to apply the inhibitors at a specific time point. It is also possible that the incomplete rescue is linked to chemical properties of the compounds. MLi-2 has a short half-life and poor aqueous solubility [52]. Consistent with this report, we observed a decrease in the solubility of MLi-2 in media incubated at 37 °C over time, indicated by precipitation of the compound in the non-homogeneous solution by absorbance measured at 620 nm (Figure S10). Given these limitations, MLi-2 might be almost completely degraded after 48 h, and any beneficial effect of the compound may have worn off by the time the movies were captured. Unfortunately, the axonal trafficking assay is technically cumbersome, making these kinds of detailed optimizations very difficult.

### 3.4. LRRK2 G2019S Is Associated with an Increase in Proportion of Lysosomes Colocalizing with Phosphorylated Rab10

Rab-GTPase proteins are well-established regulators of organelle trafficking. Rab10, a direct target of LRRK2 kinase activity, has been shown to localize to and regulate lysosomes under stress [24,26]. Since distal axons are far from the neuronal cell body, we speculated that lysosomes in distal axons might be under stress, leading to Rab10 being phosphorylated by LRRK2 at T73 (phospho-Rab10). Since the G2019S mutation increases kinase activity, neurons with LRRK2 G2019S might show an increased number of lysosomes having phospho-Rab10.

**Figure 4:**
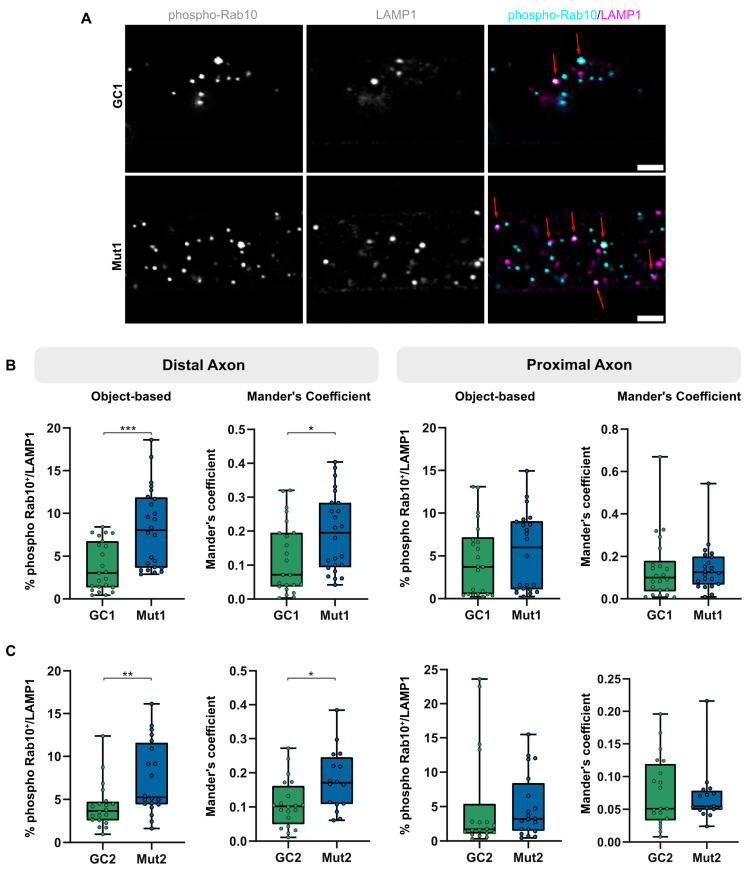
LRRK2 G2019S is associated with increased levels of phosphorylated Rab10-positive lysosomes in distal axons. (**A**) Representative micrographs of immunostaining of phosphorylated Rab10 (phospho-Rab10, cyan) and lysosomes (LAMP1, magenta) in the distal axons of Mut1 and GC1. Red arrows mark lysosomes colocalizing with phospho-Rab10. Scale bar = 2 µm (**B**) Quantification of colocalization of phospho-Rab10- and LAMP1-positive lysosomes in the distal and proximal axons of GC1 and Mut1 according to object-based analysis and Mander’s coefficient. (**C**) Quantification of colocalization of phospho-Rab10- and LAMP1-positive lysosomes in the distal and proximal axons of GC2 and Mut2 according to object-based analysis and Mander’s coefficient. For all graphs: Pooled individual measurements from N = at least three experimental replicates. Statistics were calculated using a two-tailed unpaired Mann-Whitney test. *** corresponds to *p* < 0.001, ** to *p* < 0.01, and * to *p* < 0.05.

To test this hypothesis, we assessed colocalization of LAMP1-positive lysosomes with phospho-Rab10 in the distal and proximal axons in mutant and gene-corrected isogenic neurons (Figure 4). We used object-based analysis, implemented by the JACoP ImageJ plugin [33], to identify distance between centers of objects in 3D based on their center of mass. Objects at a distance less than the resolution were marked as overlapping. Additionally, due to non-stoichiometric signal intensities in the two channels, the Mander’s coefficient was computed to estimate the proportion of LAMP1-positive lysosomes overlapping with phospho-Rab10, based on their cumulative intensity. It must be noted that LAMP1 also labels endosomes and hence LAMP1-positive structures may be a mix of endosomes and lysosomes. The overall levels of LAMP1-positive lysosomes having phospho-Rab10 were low (less than 10%), which could potentially explain why there was a relatively mild lysosomal trafficking phenotype of LRRK2 G2019S in axons. The percentage of lysosomes positive for phospho-Rab10 in the distal compartment of both Mut1 (Figure 4B, left) and Mut2 (Figure 4C, left) axons was greater than that of their respective GC1 and GC2 axons. This is particularly interesting because we observed that LRRK2 G2019S neurons showed reduced lysosomal trafficking in the distal axon, suggesting that phospho-Rab10 on distressed lysosomes may have the potential to contribute to this defect.

The increase in proportion of distal Mut1 phospho-Rab10-positive lysosomes (in comparison to its GC1) was greater than that observed in Mut2, which correlates with our axonal trafficking assay results, which detected a more reproducible lysosomal trafficking defect in Mut1 than Mut2 neurons. Colocalization of phospho-Rab10 and LAMP1 was lower in proximal axons and not significantly different between neurons with both pairs of mutant LRRK2 and isogenic controls (Figure 4B,C, right). This appears to be in agreement with our observation earlier that the retrograde lysosomal trafficking defect in proximal axons was lower than that in the distal axons and was also partially rescued on treatment with the LRRK2 inhibitor MLi-2. This suggests that low numbers of phospho-Rab10 on lysosomes may be one reason we observed such a subtle axonal trafficking defect. These LRRK2-dependent phenotypes may potentially be enhanced by increasing LRRK2 activity at a specific moment.

### 3.5. Lysosomal Membrane Damage Increases LRRK2-Mediated Rab10 Phosphorylation

Previous studies have reported that LRRK2 is activated upon lysosomal membrane damage with L-Leucyl-L-Leucine methyl ester (LLOME). LLOME is a lysosomotropic detergent that enters lysosomes via receptor-mediated endocytosis followed by conversion into a polymer that causes membrane rupture [53]. Studies using LLOME have shown activation of LRRK2 on damaged lysosomal membranes, where it recruits and phosphorylates its targets, including Rab10, initiating a series of downstream processes [24,26]. Since G2019S increases LRRK2 kinase activity, we speculated that lysosomal membrane damage might enhance LRRK2 activity and, hence, increase LRRK2-mediated Rab10 phosphorylation.

**Figure 5:**
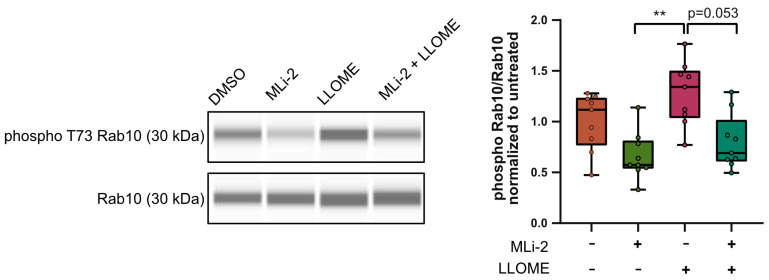
Lysosomal membrane damage increases LRRK2-mediated Rab10 phosphorylation. WES lane view of phospho T73 Rab10 and total Rab10 (**left**) with and without MLi-2 and LLOME treatments. Quantification of phospho-Rab10 levels relative to total Rab10 levels in cell lysates of Mut1 neurons with and without MLi-2 and LLOME treatments, normalized to untreated control (**right**). Pooled individual measurements from N = three experimental replicates. Statistics were calculated using the Kruskal-Wallis test with Dunn’s multiple comparisons test. ** corresponds to *p*-adj = 0.0022. (Complete uncropped WES blot in Figure S11).

To test this idea, we treated Mut1 neurons with 1 mM LLOME for 2 h to damage the lysosomal membrane. We also tested the effects of pre-treatment with 1 µM MLi-2 for 90 min before as well as during LLOME treatment. We then isolated protein from the whole cell lysate and quantified the levels of Rab10 and phospho-Rab10 using capillary electrophoresis. As expected, in comparison to DMSO, treatment with MLi-2 reduced phospho-Rab10 levels relative to total Rab10, while treatment with LLOME showed a trend toward increased relative phospho-Rab10 levels. Prior treatment with MLi-2 prevented this LLOME-induced increase in phospho-Rab10 levels (Figure 5). It should be noted that phospho-Rab10 levels were, overall, very low and at the edge of detection (Figure S11). Nevertheless, these data could indicate that endogenous LRRK2 is activated upon damage. This observation could suggest that inducing damage to the axon in a controlled fashion may reveal possible phenotypes associated with LRRK2 G2019S.

### 3.6. LRRK2 G2019S Is Not Associated with Consistent Effects on Long-Term Axonal Regrowth after Axotomy

**Figure 6:**
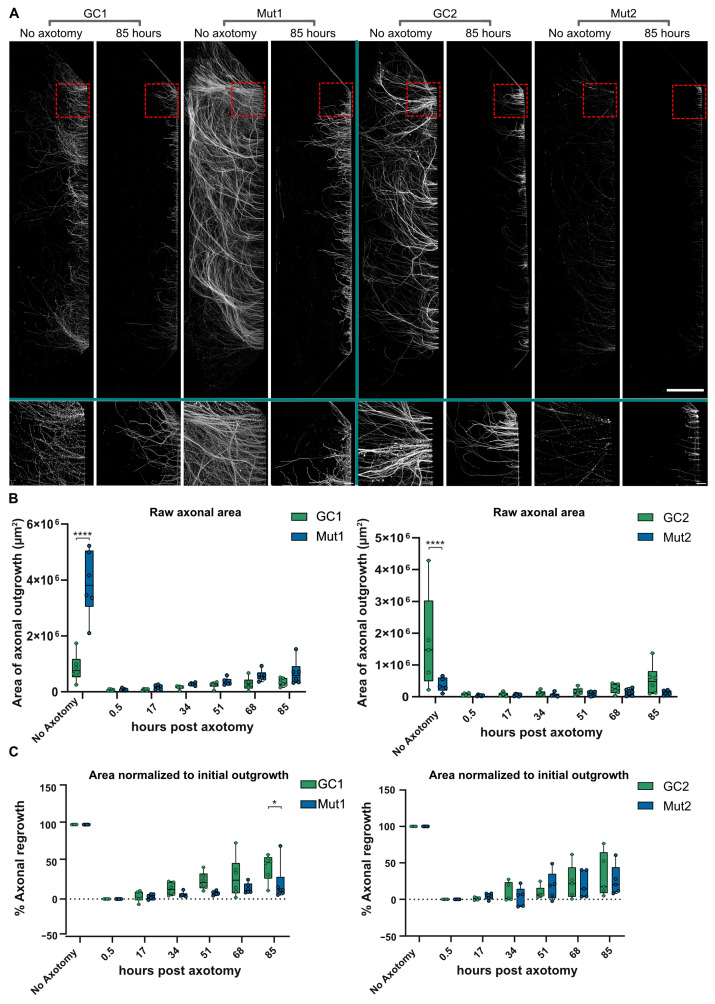
LRRK2 G2019S does not consistently affect regrowth of axons after axotomy. (**A**) Representative micrographs of TUBB3-stained GC1, Mut1, GC2, and Mut2 axons spanning the entire distal compartment (top) of separate MFCs. Conditions—no axotomy and 85 h post axotomy, with zoomed-in insets (bottom). Scale bar: 1000 µm, insets 100 µm. (**B**) Quantification of the area covered by axonal regrowth (raw data) at different times post axotomy in GC1 and Mut1 neurons (left) and GC2 and Mut2 neurons (right) (**C**) Quantification of % axonal regrowth of GC1 and Mut1 (left) and GC2 and Mut2 (right) neurons at different times post axotomy constrained between their respective no axotomy and 0.5 h controls. For all graphs: N = at least five independent experiments. Statistics were calculated using a 2-way ANOVA with Sidak’s multiple comparison test. **** corresponds to *p*-adj < 0.0001, * to *p*-adj < 0.05.

Since axons may be damaged and undergo dying back in PD, we speculated that inducing damage to the distal axon in a controlled manner may exacerbate mutant LRRK2 phenotypes. To investigate this idea, an axotomy model was utilized to introduce axonal stress in a controlled and highly localized manner. Axotomy was performed on six different chambers each in the Mut and GC lines and chambers were incubated to observe axon regrowth for 0.5, 17, 34, 51, 68, and 85 h. Afterward, lysosomes were labeled using LysoTracker and axons were immunostained for TUBB3 (Figure 6A). There was a significant difference between the area occupied by TUBB3-positive Mut and GC axons in the no axotomy controls, but there was no clear link between initial axonal growth and LRRK2 genotype (Figure 6B). Thus, to calculate axonal regrowth, each line was normalized against its respective no axotomy control. As expected, the % area of axonal regrowth did increase gradually over time (Figure 6C). Interestingly, in the first isogenic pair, GC1 axonal regrowth showed a marked increase over Mut1 at the last time point, i.e., 85 h post axotomy (Figure 6C, left panel), indicating a degree of inability of mutant axons to recover after severe axonal damage. On the other hand, Mut2 showed comparable regrowth to that of GC2 axons (Figure 6C, right panel).

Interestingly, Mut1 was also the line which showed a significant reduction in retrograde trafficking of lysosomes in our first experiments, while Mut2 showed a much softer phenotype. Additionally, according to our previous experiments, Mut1 (compared to GC1) also had a slightly greater proportion of lysosomes colocalized with phospho-Rab10 than Mut2 (compared to its isogenic control). Combining these results, it would appear that the LRRK2 G2019S mutation is relatively better tolerated in Mut2 neurons compared with in Mut1 neurons.

### 3.7. LRRK2 G2019S Is Associated with Transient Accumulation of Lysosomes at Injury Site after Axotomy

**Figure 7:**
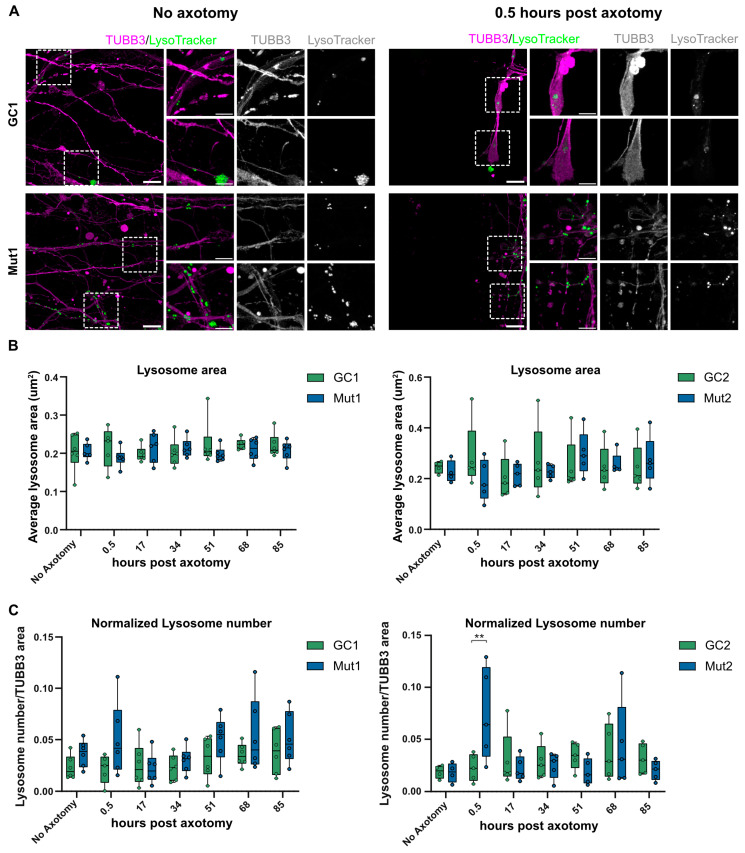
LRRK2 G2019S is associated with a transient increase in lysosome number in axons proximal to the injury site post axotomy. (**A**) Representative micrographs of LysoTracker-positive lysosomes in TUBB3-stained axons of GC1 and Mut1 neurons at the distal exit site of microchannels under the no axotomy condition (left) and 0.5 h post axotomy (right). Scale bar: 10 µm, insets 5 µm. (**B**) Quantification of the average area of lysosomes at different times post axotomy in GC1 and Mut1 neurons (left) and GC2 and Mut2 neurons (right) (**C**) Quantification of the number of lysosomes normalized to TUBB3-positive area at the distal exit site of microchannels of GC1 and Mut1 (left) and GC2 and Mut2 neurons (right) at different times post axotomy. For all graphs: N = at least five independent experiments. Statistics were calculated using a 2-way ANOVA with Sidak’s multiple comparison test. ** corresponds to *p*-adj < 0.01.

Lysosomes can be used to repair ruptures to the plasma membrane in addition to their role in protein quality control [54]. Thus, defects in axonal trafficking of lysosomes to the injury site could impact axotomy injury response. We speculated that the lysosomal trafficking defects of LRRK2 G2019S axons might interfere with lysosomal positioning after axotomy. For this reason, we quantified the LysoTracker-positive lysosomes adjacent to the axotomy injury site and normalized this to the total area of TUBB3-positive axons in the image (Figure 7A). The average area of lysosomes identified in the TUBB3-positive axons was not significantly different between the mutant and isogenic controls nor did it significantly change over time after axotomy (Figure 7B). Interestingly, in both the mutant lines, within the first 0.5 h after axotomy, there was an increase in the number of lysosomes per axonal area (Figure 7C). This phenotype was transient, as it disappeared and normalized over time. This initial phase immediately after axotomy is when the plasma membrane needs to repair and there are several reports of exocytotic lysosomes being important players in this membrane resealing [55,56]. Additionally, given that lysosomal positionality has been found to influence its pH [57], it is also possible that changes in lysosomal pH may play a role in the localization and transient increase in distal lysosomal number immediately after axotomy. Thus, our assay may be useful in identifying possible mechanistic links between PD-causing mutations such as LRRK2 G2019S and damage response in the distal axon.

### 3.8. Clinical Heterogeneity within the Donating LRRK2 G2019S Patients

**Figure 8:**
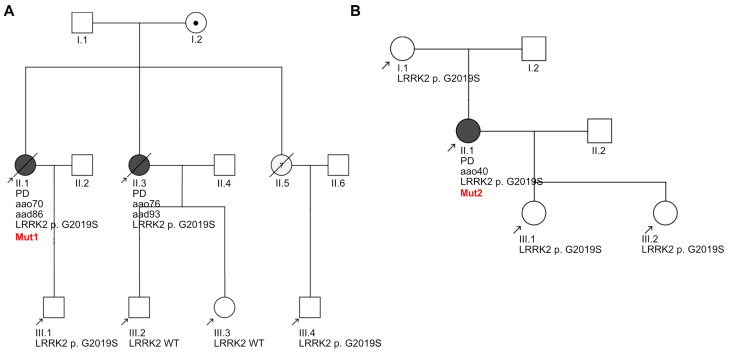
Pedigrees of the two LRRK2 G2019S-PD patients. (**A**) Pedigree of Patient1 (donor of iPSC line Mut1) and (**B**) Pedigree of Patient2 (donor of iPSC line Mut2). Symbols: square, male; circle, female; dot inside circle, mutation carrier; black-colored circles, clinically diagnosed with PD; oblique line, deceased; black arrow, individuals assessed by genetic testing. For each assessed individual, year of birth and LRRK2 status are specified. For each individual diagnosed with PD, aao indicates the age at onset and, if applicable, aad indicates the age at death.

Clinical heterogeneity has been reported among LRRK2 G2019S carriers [58], which we speculated might be linked to the heterogeneity in the phenotypes that we observed in our axonal lysosomal assays. For this reason, we sought to compare the clinical data from the two patients (Mut1 and Mut2) including their pedigrees (Figure 8).

The pedigree of Mut1 revealed that two out of the three siblings in her generation had been diagnosed with LRRK2 G2019S-PD, with two out of four individuals from the next generation carrying the mutation (Figure 8A). Born in 1931, the age at onset of this patient was 70 years, and biomaterial collection for iPSC generation was performed at 77 years. The pedigree of the second patient, Mut2, indicates that she inherited the LRRK2 G2019S mutation from her mother, who did not develop PD (Figure 8B). Individuals from the next generation are carriers of the mutation, indicating that it is familial in nature. Born in 1958, this individual developed early-onset PD at the age of 40 years and biomaterial for iPSC generation was collected at 50 years. Analysis of genetic data from the two patient-specific iPSCs (Table S2) showed that Mut1 was heterozygous for the “at risk” *MAPT* H1 haplotype and homozygous for “disease protective” *SNCA* polymorphism rs356220. Mut2 was identified to be homozygous for both at risk *MAPT* and *SNCA* polymorphisms. Neither of the patients had the Alzheimer’s APOE risk allele E4.

According to the clinical data collected over several outpatient clinic visits (Table S3), Mut1 had a much lower Levodopa-equivalent daily dosage (LEDD) than Mut2 and showed a more advanced stage of PD according to H&Y scoring [43] and UPDRS-III rating [42]. According to their MoCA score [44], Mut1 was also diagnosed with mild cognitive impairment, which had progressed to dementia before their last visit. Mut2 showed no cognitive impairment as reported by their MoCA score even up to her last clinic visit—a disease duration of 24 years. This correlated with CSF levels of Aβ1–42, total Tau, and phosphorylated Tau (T181), which are a sign of cognitive decline or dementia in some PD patients [59]: Mut1 presented with lower levels of Aβ1–42 and higher levels of total and phosphorylated Tau, while levels of these biomarkers in Mut2 were detected in the normal range according to lab cutoffs (Aβ1–42 < 599 pg/mL, total Tau > 404 pg/mL, and phosphorylated Tau > 56.5 pg/mL). CSF from both patients tested positive in the α-synuclein seed amplification assay, which detects Lewy pathology [60]. CSF NfL levels were also found to be higher in Mut1 than Mut2, indicating a more severe phenotype; however, levels of NfL have also been shown to increase with age [61]. Overall, these results indicated that the two LRRK2 G2019S-PD patients were very distinct, with the Mut1 patient showing a more advanced PD stage with motor and cognitive decline than Mut2, indicating involvement of several neuronal cell populations. It is interesting to note that the axonal lysosomal trafficking phenotypes in Mut1 neurons were observed with both methods of analysis while those in Mut2 neurons was observed in distal axons with only one analysis method. This suggests that our assays could be useful for characterizing the molecular heterogeneity contributing to PD heterogeneity. Data from additional patient lines would be needed to assess disease-phenotype correlations.

## 4. Discussion

PD neuronal axons die back several years before a clinical diagnosis. By the time symptoms begin to appear, about 50–70% of the axonal terminals are lost [9]. LRRK2 patients show a similar trend. A cohort of 25 asymptomatic LRRK2 G2019S carriers showed gradual decline of dopaminergic neuron terminals via DaT scan (^123^I-ioflupane SPECT) over 8 years [62]. Thus, assays focusing on axonal pathology are urgently needed to better understand and protect against dying back. Since trafficking of lysosomes is responsible for their timely presence at the required sites of action and then migration back to the soma for maturation, we hypothesized that studying lysosomal trafficking in the axons of iPSC-derived neurons from patients may provide insights into the early pathogenesis of PD. We focused on LysoTracker-positive vesicles, which are reported to stain acidic compartments including lysosomes, but potentially also other vesicle populations. While setting up the axonal trafficking assay, we observed that small changes in the analysis pipeline tended to influence the results. This called for comparing outputs from independent algorithms to reach a more confident conclusion. We found that the retrograde trafficking of lysosomes was reduced in the LRRK2 G2019S axons compared to isogenic controls. Our results tracking lysosomes are along the similar lines of a report showing that mutations in LRRK2 disrupt autophagosome transport [63], indicating possibly wider effects of LRRK2 mutations on the autophagosomal-lysosomal system. However, this was a pilot study that used only two patient-derived lines to develop its methodology, and further analysis using additional iPSC lines is needed. Thus, our assay could be useful in identifying early axonal phenotypes associated with PD risk alleles.

Another interesting point to consider is that we observed extremely small differences in the speed of lysosomes in our in vitro assays. This was observed over a distance of merely 117.53 µm (field of view). Additionally, the region that we refer to as distal axons is at best merely 782.47 µm away from the proximal axons (subtracting the field of view of proximal axons = 117.53 µm from the length of microchannels = 900 µm). It is likely that further differences between distal and proximal axons may emerge if we were to increase the distance between the two readout positions to focus on truly “proximal” or “distal” axons. Intriguingly, if we extrapolate our findings to the in vivo length of the total arborization tree of human dopaminergic axons that is estimated to be upwards of 4 m [64,65], the consequences of the small change in speed we observed is likely to be profound over the lifetime of the individual.

PD is clinically very heterogeneous, and LRRK2 G2019S is exemplary of this, manifesting as both early- and late-onset PD, and presents with a wide range of clinical symptoms which could be caused by diverse pathophysiological features. The two patient donors of our cells reflected this heterogeneity and had distinct clinical profiles. Mut1 developed late-onset PD with severe motor symptoms and reduced cognitive abilities. In contrast, Mut2 developed early-onset PD with relatively less severe motor symptoms and normal cognitive abilities. Interestingly, we found that Mut1 neurons and Mut2 neurons also manifested distinct phenotypes, and it is tempting to speculate that these differences may be linked. However, these differences could also be due to other effects, such as stochastic clonal differences. Applying our assays to iPSC-derived neurons from additional patients could be a step toward establishing disease subtype-specific readouts, which could enable the testing of subtype-specific therapeutics.

Since LRRK2 G2019S has been shown to increase its kinase activity, we tested if LRRK2 inhibitors would rescue the axonal trafficking defect observed in the mutant line. Since Mut1 showed the strongest trafficking phenotype, we tested the effects of treatment of Mut1 neurons with LRRK2 inhibitors CZC-25146 or MLi-2. We observed a partial increase in lysosomal speed with MLi-2. Although CZC-25146 showed no effect, MLi-2 is about 10 times more potent. However, we did not observe a complete rescue of lysosomal speed on treatment with MLi-2. One possible explanation is that LRRK2 G2019S is not the only factor contributing to the axonal trafficking defect. Consistent with this idea, both Mut1 and Mut2 neurons show additional genetic risk polymorphisms, including the *MAPT* H1 haplotype and *SNCA* rs356220 SNP. It is also important to note that axonal trafficking is a “chronic” phenotype that lacks a clear beginning and end, making it difficult to identify the ideal time points for rescue using a small-molecule drug. Additionally, it has been reported for other neurodegenerative diseases that striking axonal trafficking defects are observed in neurons kept in culture for longer timelines—more than 60 days after seeding in MFC [66]—which could possibly inform the best time point of analysis. Our assays, which impart a controlled axonal stress, may enable detection of LRRK2-associated phenotypes. Interestingly, both the genetic and timing issues hold critical implications for selecting patients and refining treatment strategies in clinical trials of the kinase inhibitors. For this reason, our assays may provide informative information when testing novel protective therapeutics.

## 5. Conclusions

PD is characterized by dying back, and this study establishes assays for multiple distal axon phenotypes using iPSC-derived neurons. We show that there is a subtle lysosomal trafficking phenotype that was partially rescued by a LRRK2 inhibitor. Mutant LRRK2 neurons showed increased phosphorylated Rab10-positive lysosomes, and damaging the lysosomal membrane increased LRRK2-dependent Rab10 phosphorylation. Neurons with mutant LRRK2 showed a transient increase in lysosomes at axotomy injury sites. These subtle phenotypes reveal preliminarily interesting effects of LRRK2 G2019S and warrant analysis with additional neuronal lines. Therefore, our axonal lysosomal assays can potentially be used to characterize early PD pathogenesis in additional lines and test therapeutics.

## Data Availability

Analysis pipelines are deposited here: https://figshare.com/s/0a68d2bbe3a84e11ff0e Accessed on 23 October 2023.

## Supplementary Materials

The following supporting information can be downloaded at: https://www.mdpi.com/article/10.3390/biology13010058/s1, Figure S1: Expression of neural progenitor markers in NPCs and neuronal markers in differentiated neurons; Figure S2: Spatial isolation of axons from soma and dendrites using microfluidic chambers; Figure S3: Directionality of lysosomes; Figure S4: Quantification of mean speed of lysosomal trafficking in GC1 and Mut1 neurons; Figure S5: Quantification of mean speed of lysosomal trafficking in GC2 and Mut2 neurons; Figure S6: LRRK2 G2019S is associated with a small reduction in the displacement of retrograde lysosomal trafficking in Mut1 neurons; Figure S7: LRRK2 G2019S is not associated with a consistent change in the displacement of lysosomal trafficking in Mut2 neurons; Figure S8: LRRK2 kinase inhibitors do not significantly affect the displacement of retrograde lysosomes in the distal or proximal axon; Figure S9: LRRK2 inhibitor treatment does not affect the speed of Mut2 axonal lysosomes; Figure S10: Precipitation of MLi-2 in media incubated at 37 °C over time; Figure S11: Uncropped WES blots of phospho-Rab10 and total Rab10 levels in LLOME and MLi-2 treated Mut1 neurons; Table S1: Summary statistics and p-adj-values for axonal trafficking data; Table S2: Genetic information of the two LRRK2 G2019S-PD patient donors; Table S3: Demographic, clinical, and CSF biomarker information of the two LRRK2 G2019S-PD patient donors.
