# Supplementary material for: Axonal Lysosomal Assays for Characterizing the Effects of LRRK2 G2019S
Source: Biology (Basel). 2024 Jan 20;13(1):58. doi: 10.3390/biology13010058 (PMC10813644; doi:10.3390/biology13010058)
Supplement: Supplementary file 1 [file biology-13-00058-s001.zip › biology-2739774-supplementary.pdf]

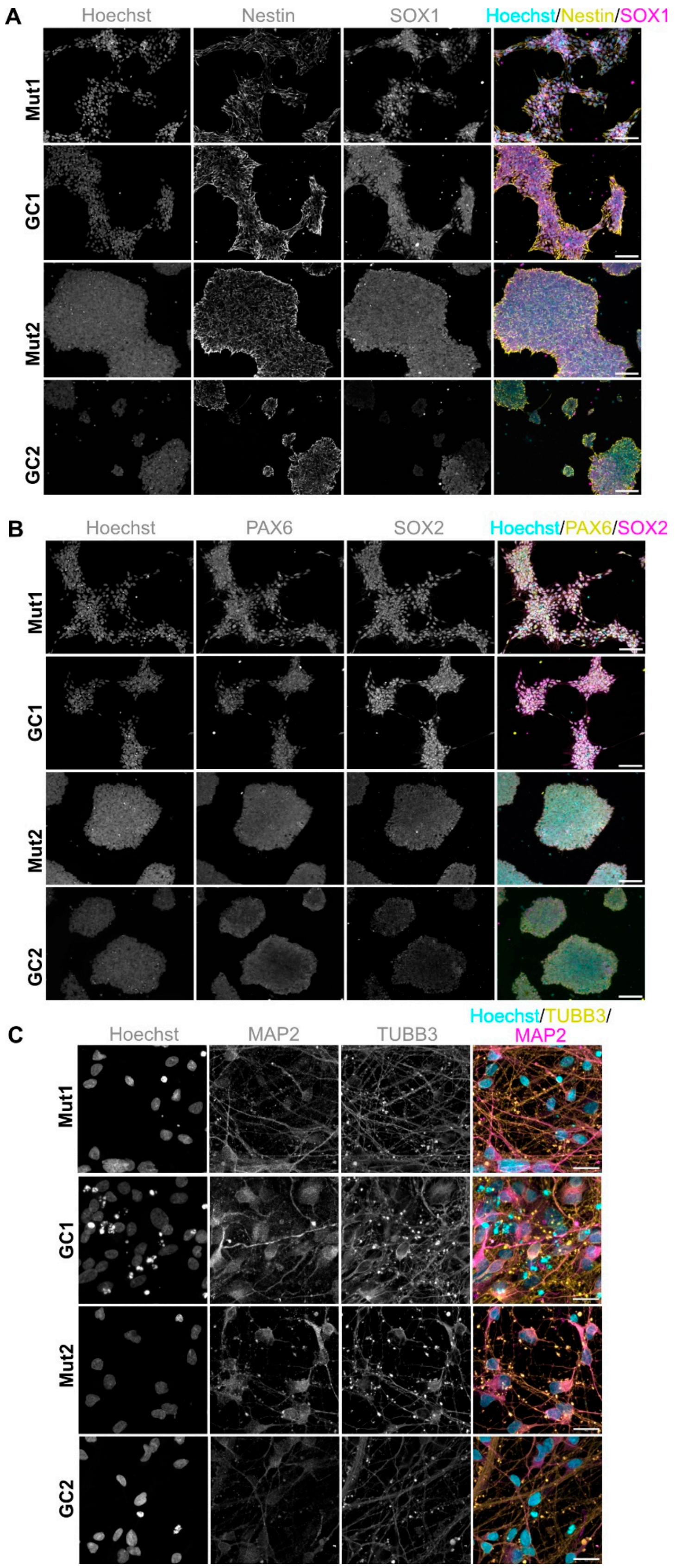
A
Hoechst
Nestin
SOX1
Hoechst/Nestin/SOX1
Mut1
GC1
Mut2
GC2
B
Hoechst
PAX6
SOX2
Hoechst/PAX6/SOX2
Mut1
GC1
Mut2
GC2
C
Hoechst
MAP2
TUBB3
Hoechst/TUBB3/MAP2
Mut1
GC1
Mut2
GC2

Figure S1: Expression of neural progenitor markers in NPCs and neuronal markers in differentiated neurons. Representative micrographs confirm the presence of neural progenitor markers **(A)** Nestin and SOX1, **(B)** PAX6 and SOX2 in Mut1, GC1, Mut2, and GC2. Scale bar = 100 µm. **(C)** Representative micrographs confirm the presence of pan-neuronal markers MAP2 and TUBB3 in neurons differentiated from Mut1, GC1, Mut2, and GC2. Scale bar = 25 µm.

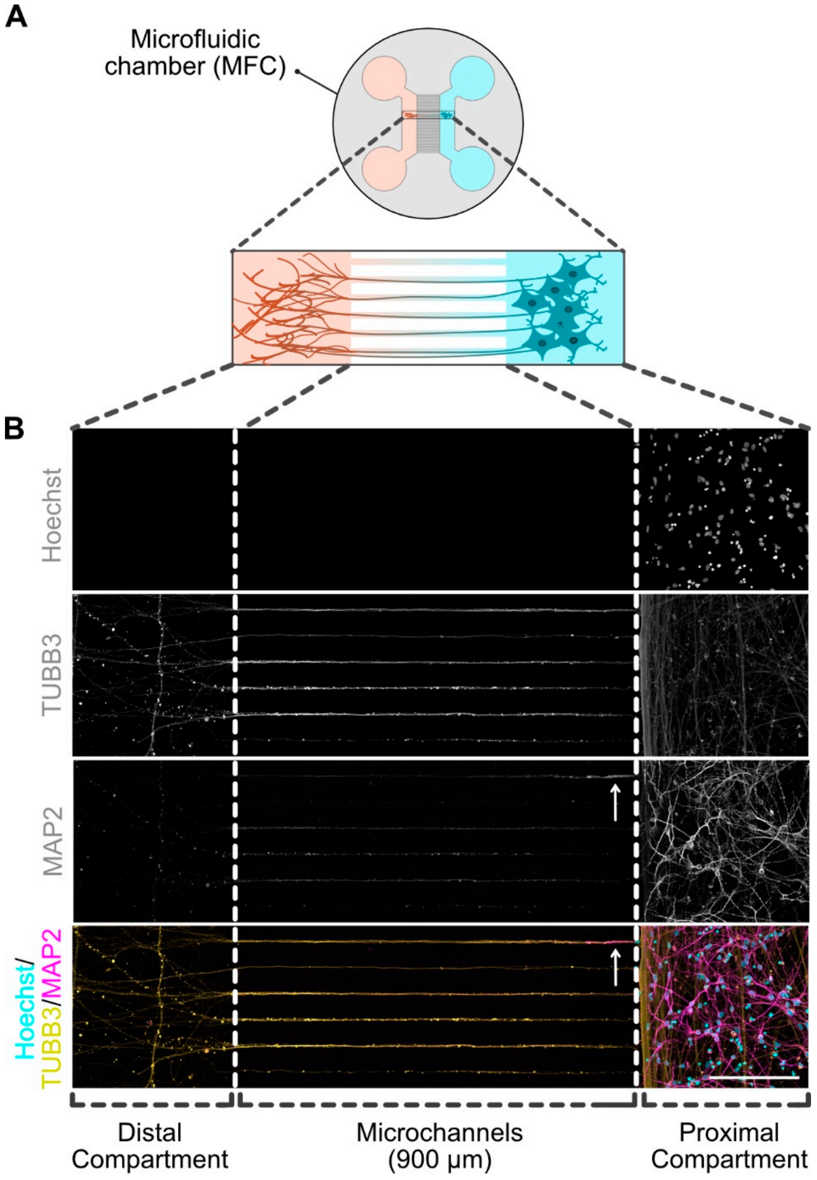

Figure S2: Spatial isolation of axons from soma and dendrites using microfluidic chambers.
(A) Schematic representation of MFCs, with soma in the proximal compartment and axonal outgrowth through microchannels (900 µm) to the distal compartment. **(B)** Representative micrographs confirm TUBB3-positive axons in the distal compartment, while nuclei (Hoechst) and dendrites (MAP2) remain primarily in the proximal compartment. A white arrow marks the initial section of a microchannel penetrated by dendrites. Scale bar = 200 µm.

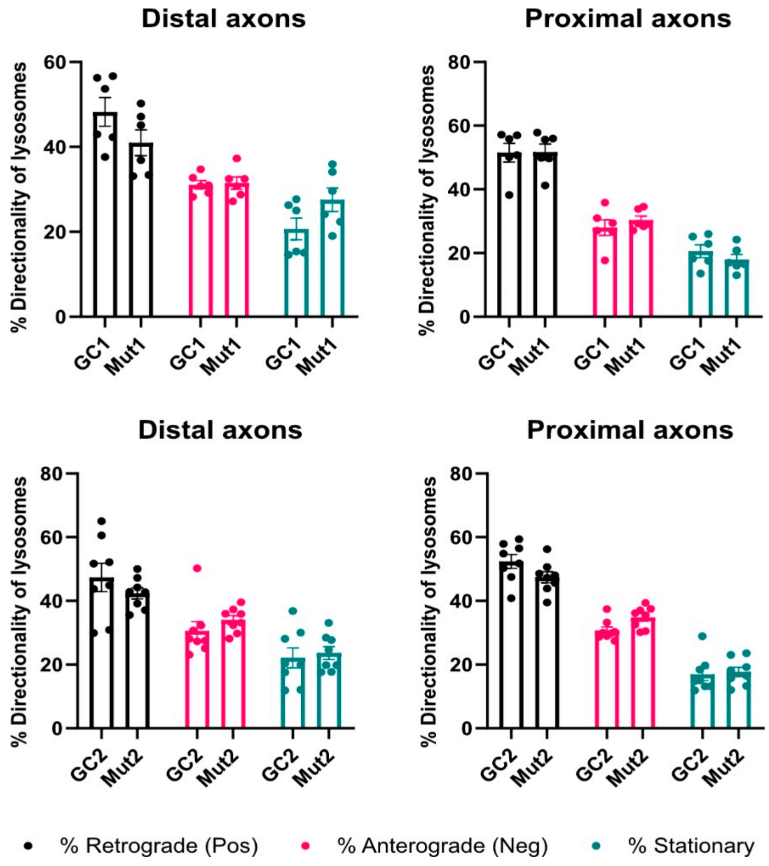

Figure S3: Directionality of lysosomes.
The relative proportion of retrograde, anterograde, and stationary lysosomes in distal and proximal axons of GC1-Mut1 (top) and GC2-Mut2 (bottom). There is no significant difference between GC and Mut in any direction, according to 2-way ANOVA.

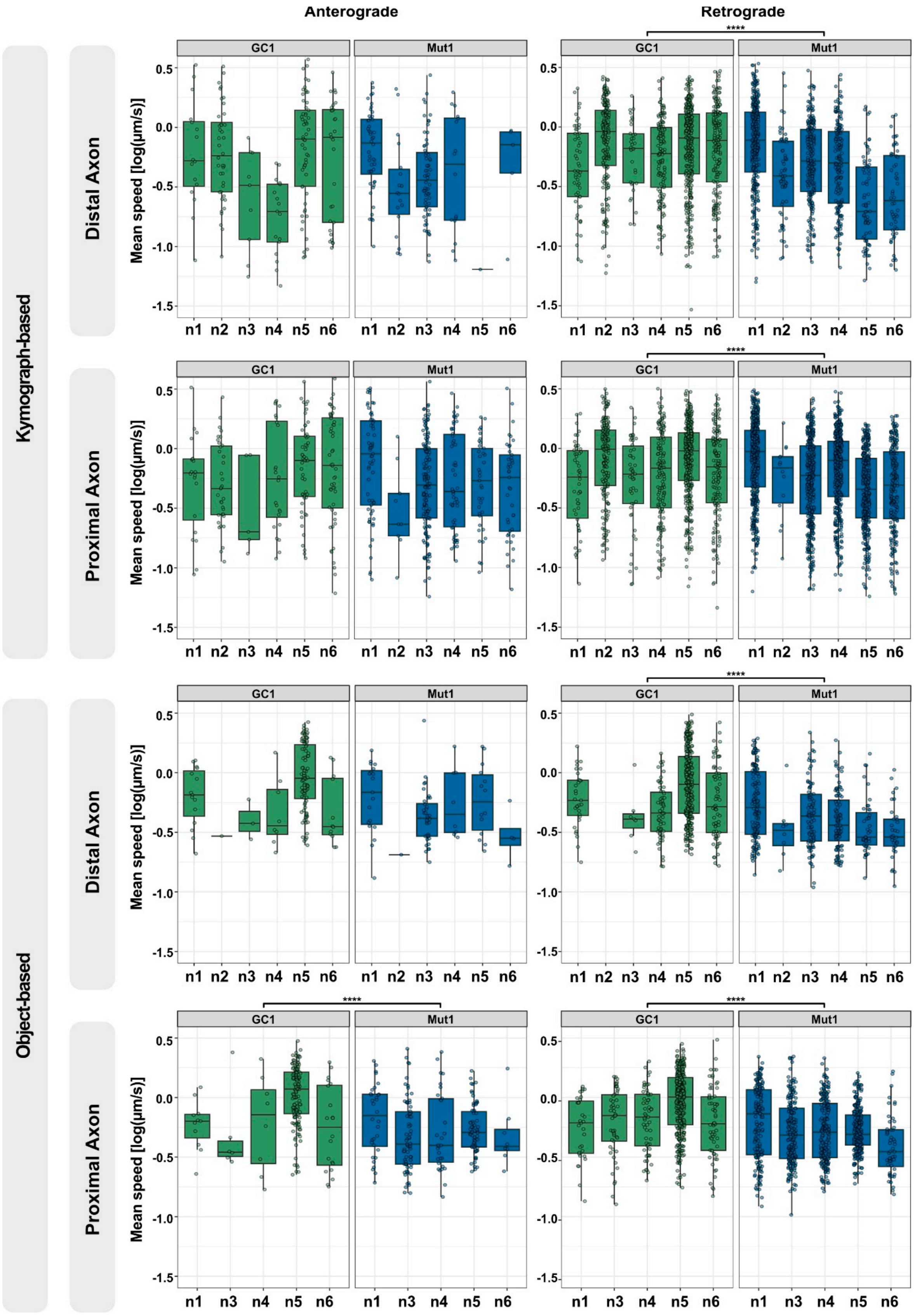

Anterograde
Retrograde
Kymograph-based
Object-based
Distal Axon
Proximal Axon
GC1
Mut1
****
Mean speed [log(μm/s)]
0.5
0.0
-0.5
-1.0
-1.5
n1 n2 n3 n4 n5 n6
n1 n3 n4 n5 n6

Figure S4. Quantification of mean speed of lysosomal trafficking in GC1 and Mut1 neurons.
Modified graphical representation of Figure 2A, with box-plots of individual experimental replicates (n), quantifying the mean speed of anterograde and retrograde lysosomes in distal and proximal axons of GC1 and Mut1 neurons by kymograph-based analysis (top) and object-based analysis (bottom). Statistics were calculated using 2-way ANOVA with post-hoc Tukey test to compare GC and Mut, considering any variation between the experimental replicates. **** corresponds to p-adj <= 0.0001. Comparisons without a marked * did not report any significant differences. The exact mean difference and p-values can be found in Table S1.

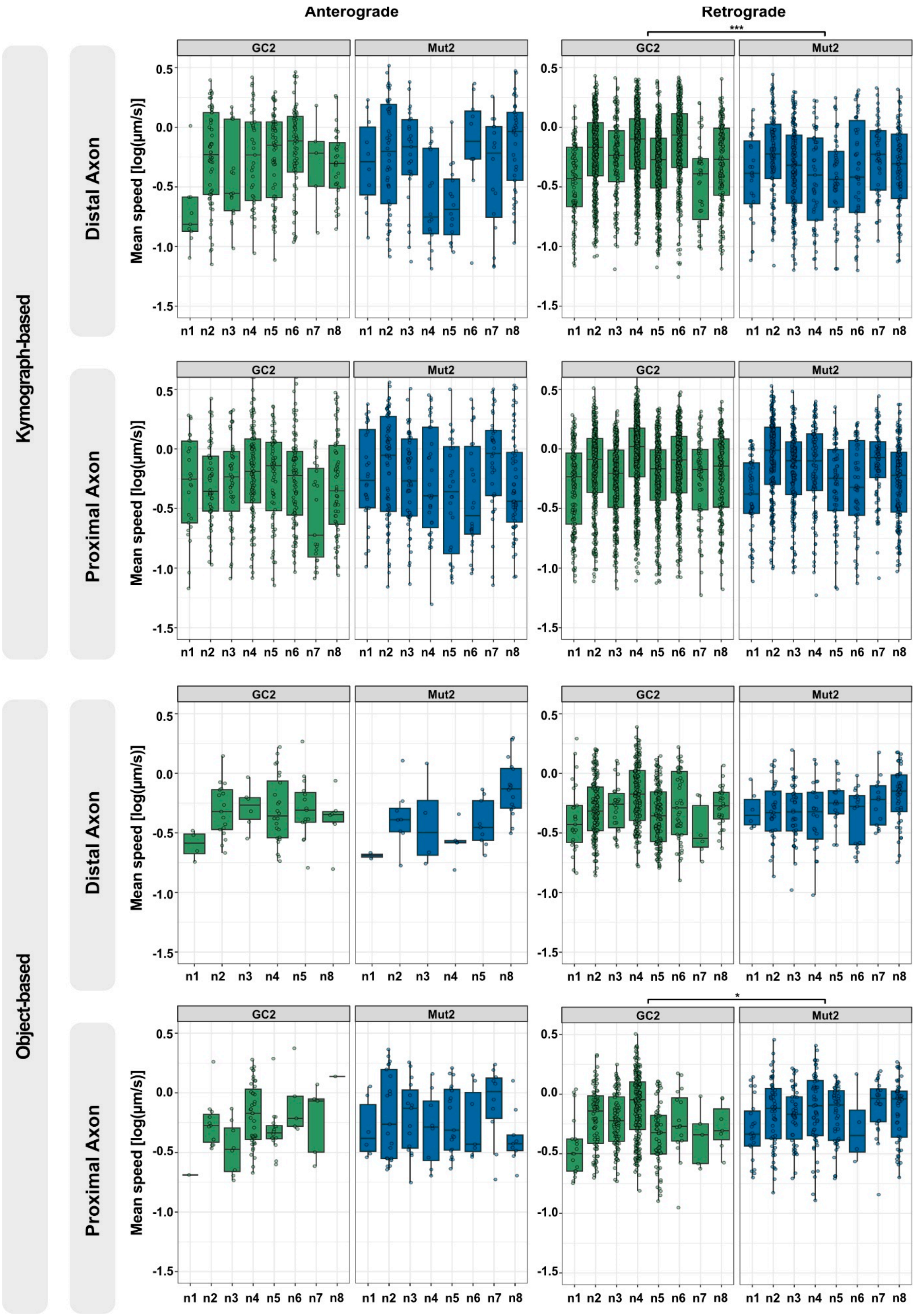

Anterograde
Retrograde
***
*
GC2
Mut2
Kymograph-based
Object-based
Distal Axon
Proximal Axon
Mean speed [log(µm/s)]
0.5
0.0
-0.5
-1.0
-1.5
n1 n2 n3 n4 n5 n6 n7 n8
n1 n2 n3 n4 n5 n8
n1 n2 n3 n4 n5 n8

Figure S5. Quantification of mean speed of lysosomal trafficking in GC2 and Mut2 neurons.
Modified graphical representation of Figure 2B, with box-plots of individual experimental replicates (n), quantifying the mean speed of anterograde and retrograde lysosomes in distal and proximal axons of GC1 and Mut1 neurons by kymograph-based analysis (top) and object-based analysis (bottom). Statistics were calculated using 2-way ANOVA with post-hoc Tukey test to compare GC and Mut, considering any variation between the experimental replicates. *** corresponds to p-adj <= 0.001, and * to p-adj <= 0.05. Comparisons without a marked * did not report any significant differences. The exact mean difference and p-values can be found in Table S1.

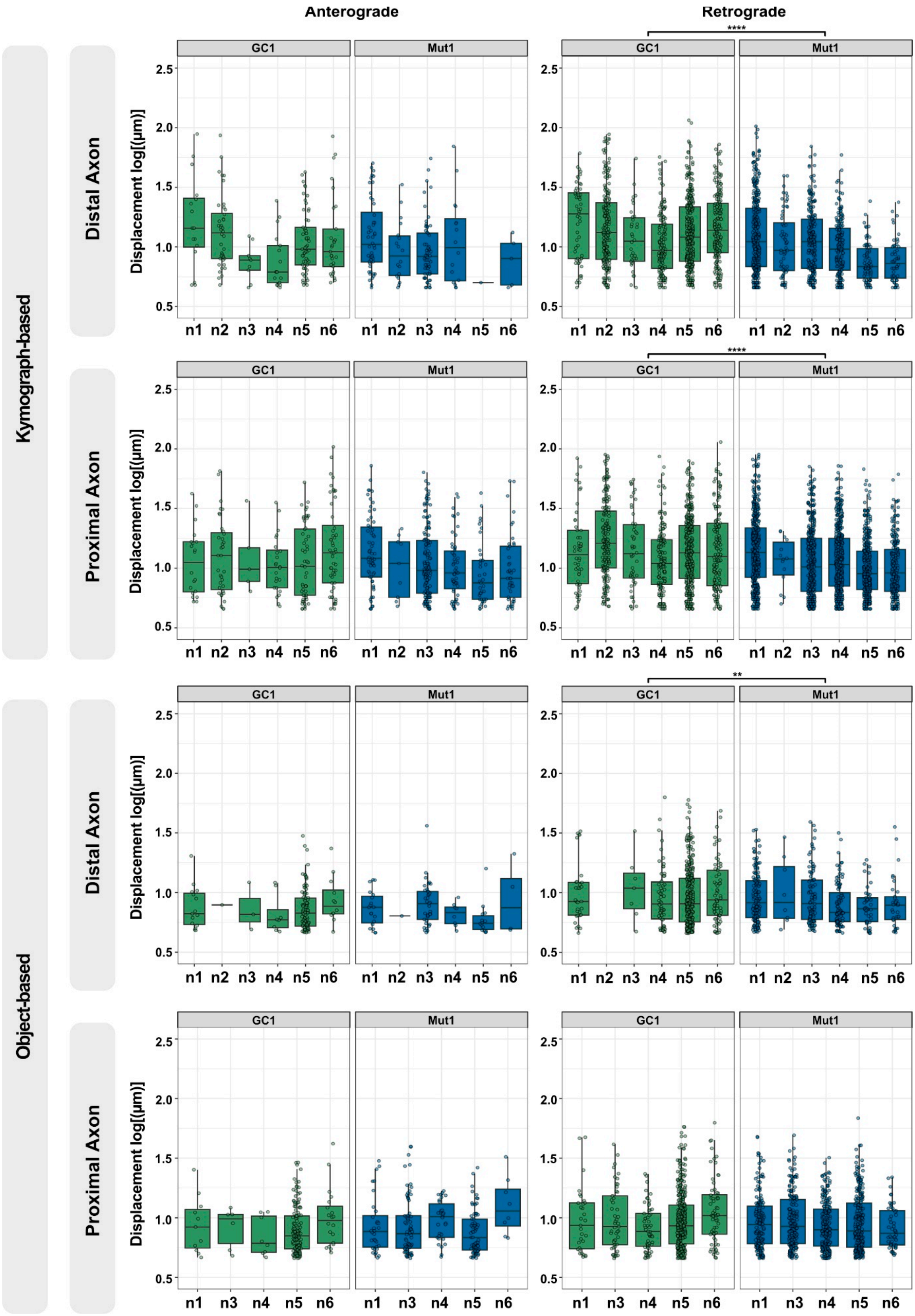

Anterograde
Retrograde
Kymograph-based
Object-based
Distal Axon
Proximal Axon
GC1
Mut1
****
**
Displacement log[(µm)]
2.5
2.0
1.5
1.0
0.5
n1 n2 n3 n4 n5 n6
n1 n3 n4 n5 n6

Figure S6: LRRK2 G2019S is associated with a small reduction in the displacement of retrograde lysosomal trafficking in Mut1 neurons.

Quantification of displacement of anterograde and retrograde lysosomes in the distal and proximal axons of GC1 and Mut1 neurons by kymograph-based analysis (top) and object-based analysis (bottom). Statistics were calculated using 2-way ANOVA with post-hoc Tukey test to compare GC and Mut, taking into consideration any variation between the experimental replicates (n). **** corresponds to p-adj <= 0.0001, ** to p-adj <= 0.01. Comparisons without a marked * did not report any significant differences. The exact mean difference and p-values can be found in Table S1.

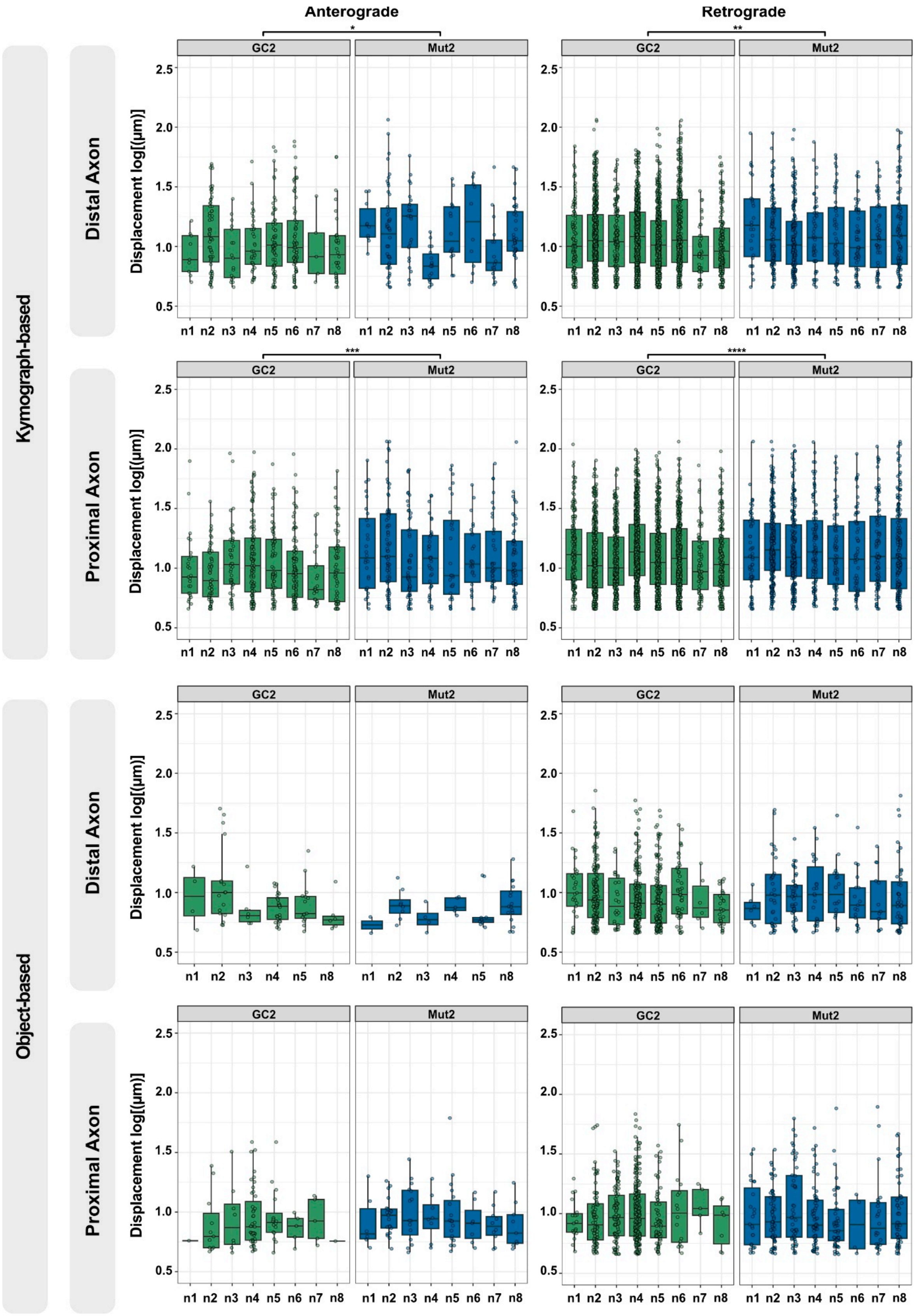

Anterograde
Retrograde
Kymograph-based
Object-based
Distal Axon
Proximal Axon
GC2
Mut2
Displacement log[(µm)]
n1 n2 n3 n4 n5 n6 n7 n8
*
**
***
****

Figure S7: LRRK2 G2019S is not associated with a consistent change in the displacement of lysosomal trafficking in Mut2 neurons.

Quantification of displacement of anterograde and retrograde lysosomes in the distal and proximal axons of GC2 and Mut2 neurons by kymograph-based analysis (top) and object-based analysis (bottom). Statistics were calculated using 2-way ANOVA with post-hoc Tukey test to compare GC and Mut, taking into consideration any variation between the experimental replicates (n). **** corresponds to p-adj <= 0.0001, *** to p-adj <= 0.001, ** to p-adj <= 0.01, and * to p-adj <= 0.05. Comparisons without a marked * did not report any significant differences. The exact mean difference and p-values can be found in Table S1.

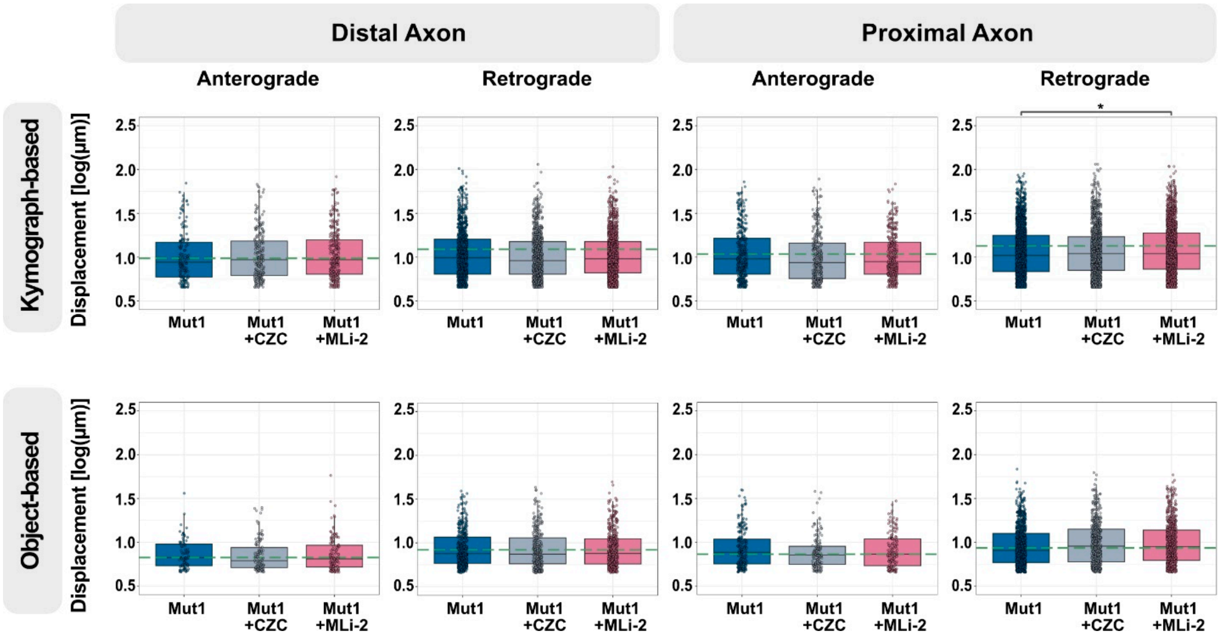

Figure S8: LRRK2 kinase inhibitors do not significantly affect the displacement of retrograde lysosomes in the distal or proximal axon.

Quantification of displacement of anterograde and retrograde lysosomes in the distal and proximal axons of Mut1 neurons without and with 2 µM LRRK2 kinase inhibitors CZC-25146 or MLi-2 for 48 hours by kymograph-based analysis (top) and object-based analysis (bottom). The green dashed line marks the median lysosomal displacement of GC1 in the specific direction and position. For all graphs: Pooled individual measurements from N = at least 6 independent experimental replicates. Statistics were calculated using 2-way ANOVA with post-hoc Tukey test to compare the effect of compound treatment, taking into consideration any variation between the experimental replicates. * corresponds to p-adj <= 0.05. Comparisons without a marked * were not significantly different than Mut1. The exact mean difference and p-values can be found in Table S1.

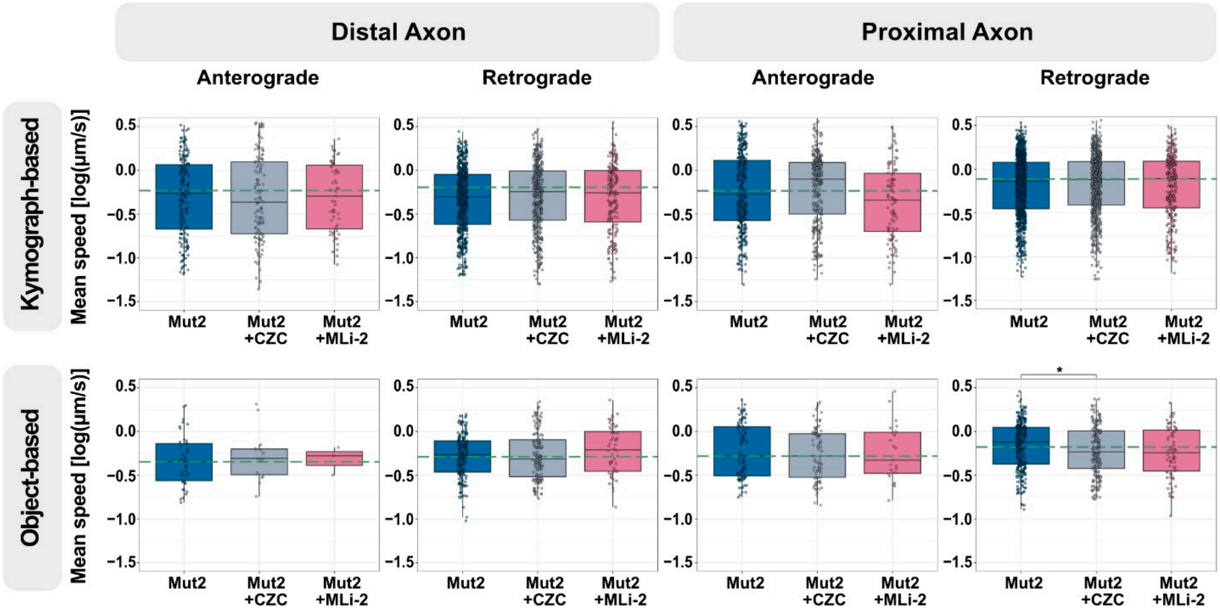

Figure S9: LRRK2 inhibitor treatment does not affect the speed of Mut2 axonal lysosomes.
Quantification of the mean speed of anterograde and retrograde lysosomes in the distal and proximal axons of Mut2 neurons without and with 2 µM LRRK2 kinase inhibitors CZC-25146 or MLi-2 for 48 hours by kymograph-based analysis (top) and object-based analysis (bottom). The green dashed line marks the median lysosomal speed of GC2 in the specific direction and position. For all graphs: Pooled individual measurements from N = at least 6 independent experimental replicates. Statistics were calculated using 2-way ANOVA with post-hoc Tukey test to compute the effect of compound treatment on comparison with no treatment, taking into consideration any variation between the experimental replicates. * corresponds to p-adj <= 0.05. Comparisons without a marked * were not significantly different than Mut2. The exact mean difference and p-values can be found in Table S1.

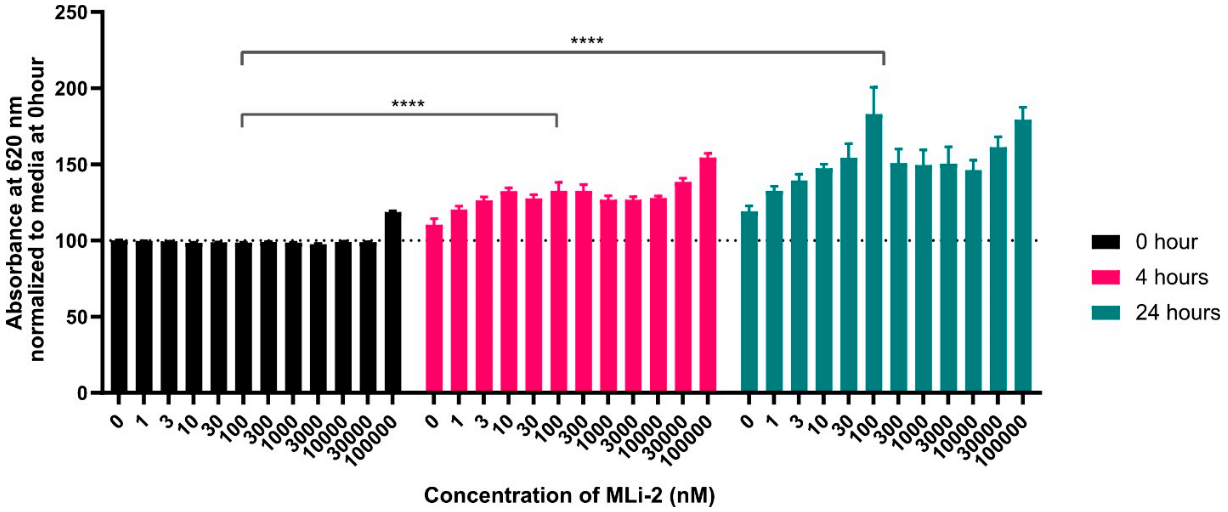

Figure S10: Precipitation of MLi-2 in media incubated at 37°C over time.

MLi-2 diluted in maturation medium over a range of concentrations was incubated at 37°C for 24 hours. Turbidimetric solubility assay measuring the absorbance at 620 nm at 0, 4, and 24 hours revealed the onset of precipitation and lack of solubility of MLi-2 over time based on the increase in absorbance value with MLi-2 relative to that of only media control at the start of the experiment. Statistics were calculated using 2-way ANOVA with post-hoc Tukey's multiple comparisons test. **** corresponds to p-adj<= 0.0001.

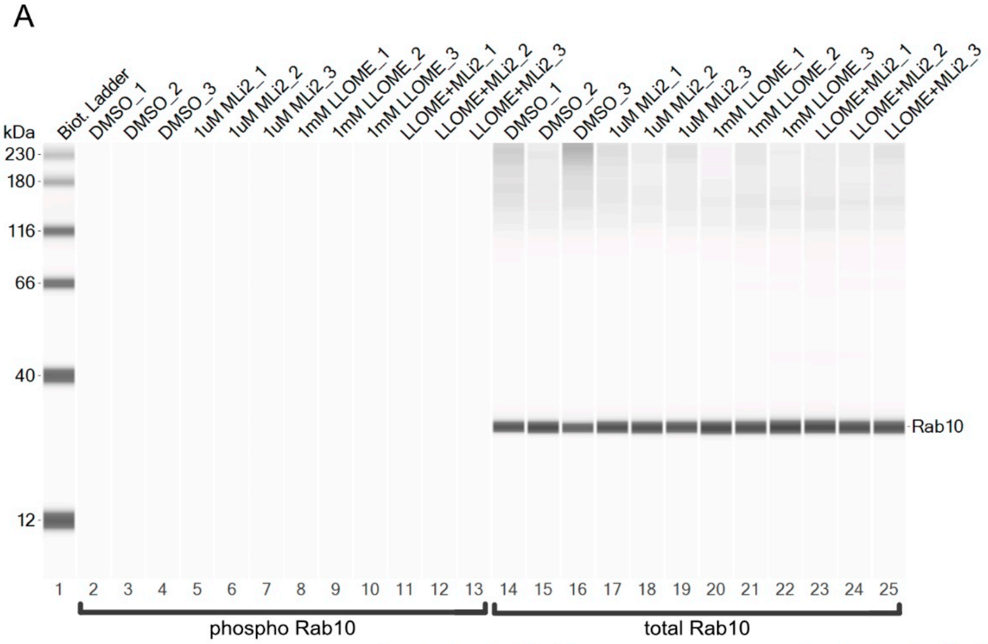

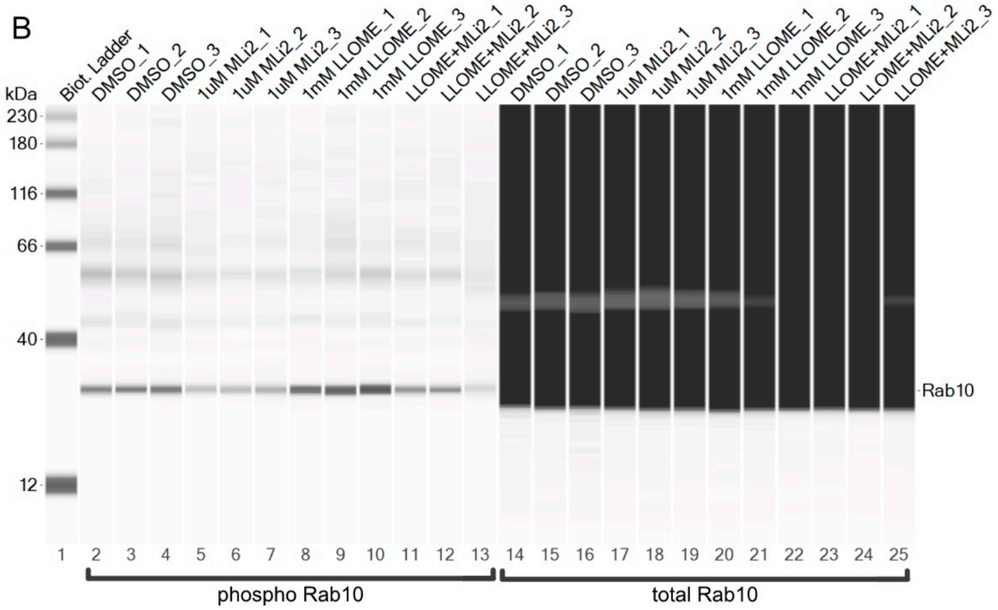

Figure S11: Uncropped WES blots of phospho-Rab10 and total Rab10 levels in LLOME and MLi-2 treated Mut1 neurons.

Uncropped Wes lane view (from Figure 5) measuring levels of phospho Rab10 and total Rab10 from whole cell lysates of DMSO, MLi-2, LLOME and MLi-2+LLOME treated Mut1 neurons (3 technical replicates for each condition). **(A)** Exposed Lanes 14-25 detecting the 30kDa total Rab10. **(B)** Adjusted contrast to detect phospho-Rab10 in Lanes 2-13.

Figure No.	Position	Direction	Parameter	Analysis method	Condition1	Condition 2	Mean difference	p-adj-value	95% confidence interval	
									Lower bound	Upper bound
2A	Distal	Retrograde	Mean speed [log(um/s)]	Kymograph-based	GC1	Mut1	0.118901234	1.4E-11	0.085709452	0.152093017
2A	Distal	Retrograde	Mean speed [log(um/s)]	Object-based	GC1	Mut1	0.107607569	1.69E-08	0.070534005	0.144681133
2A	Proximal	Anterograde	Mean speed [log(um/s)]	Object-based	GC1	Mut1	0.141639852	3.25E-07	0.0880848	0.195194904
2A	Proximal	Retrograde	Mean speed [log(um/s)]	Kymograph-based	GC1	Mut1	0.072461235	1.45E-07	0.045506107	0.099416362
2A	Proximal	Retrograde	Mean speed [log(um/s)]	Object-based	GC1	Mut1	0.138734883	0	0.110857264	0.166612503
2B	Distal	Retrograde	Mean speed [log(um/s)]	Kymograph-based	GC2	Mut2	0.074935882	1.18692E-06	0.044754377	0.105117387
2B	Proximal	Retrograde	Mean speed [log(um/s)]	Object-based	GC2	Mut2	-0.046588629	0.014573529	-0.08394361	-0.00923365
S4A	Distal	Retrograde	Displacement [log(um)]	Kymograph-based	GC1	Mut1	0.088654611	4.38E-11	0.062606388	0.114702834
S4A	Distal	Retrograde	Displacement [log(um)]	Object-based	GC1	Mut1	0.040756978	0.009466613	0.009997132	0.071516823
S4A	Proximal	Retrograde	Displacement [log(um)]	Kymograph-based	GC1	Mut1	0.074596169	0	0.052226137	0.096966201
S4B	Distal	Anterograde	Displacement [log(um)]	Kymograph-based	GC2	Mut2	-0.056465741	0.037228959	-0.10957542	-0.00335606
S4B	Distal	Retrograde	Displacement [log(um)]	Kymograph-based	GC2	Mut2	-0.035949562	0.007478874	-0.06228446	-0.00961467
S4B	Proximal	Anterograde	Displacement [log(um)]	Kymograph-based	GC2	Mut2	-0.083745168	0.000151062	-0.12691869	-0.04057164
S4B	Proximal	Retrograde	Displacement [log(um)]	Kymograph-based	GC2	Mut2	-0.065969888	1.93E-08	-0.08891582	-0.04302396
3	Distal	Retrograde	Mean speed [log(um/s)]	Kymograph-based	Mut1	Mut1+MLi-2	-0.039556723	0.032508707	-0.07651532	-0.00259813
3	Proximal	Retrograde	Mean speed [log(um/s)]	Kymograph-based	Mut1	Mut1+MLi-2	-0.046533139	0.000138543	-0.07330616	-0.01976012
3	Proximal	Retrograde	Mean speed [log(um/s)]	Object-based	Mut1	Mut1+MLi-2	-0.073381438	5.51E-08	-0.10384618	-0.0429167
S5	Proximal	Retrograde	Displacement [log(um)]	Kymograph-based	Mut1	Mut1+MLi-2	-0.023244226	0.036452445	-0.04533914	-0.00114931
S6	Proximal	Retrograde	Mean speed [log(um/s)]	Object-based	Mut2	Mut2+CZC	0.066739437	0.022940438	0.00740384	0.126075033
3	Proximal	Retrograde	Mean speed [log(um/s)]	Kymograph-based	GC1	Mut1+CZC	0.08083407	2.71E-07	0.042904559	0.118763581
3	Proximal	Retrograde	Mean speed [log(um/s)]	Object-based	GC1	Mut1+CZC	0.122714657	0	0.082222893	0.163206421
3	Proximal	Retrograde	Mean speed [log(um/s)]	Kymograph-based	GC1	Mut1+MLi-2	0.033603105	0.070854058	-0.00186831	0.069074517
3	Proximal	Retrograde	Mean speed [log(um/s)]	Object-based	GC1	Mut1+MLi-2	0.085325138	4.28E-08	0.047540621	0.123109655

Table S1: Summary statistics and p-adj-values for axonal trafficking data

Mean difference, adjusted p-values, lower and upper bound of the confidence interval for the mean, resulting from 2-way ANOVA and Tukey's multiple comparisons test. Only comparisons reported as statistically significant in each of the listed conditions are specified.

**Pat ID**	**APOE Haplotype**	***MAPT* Haplotype (H2=protective, H1=risk)**	***SNCA* rs356220 or proxy rs356219 (C=protective, T=risk)**	**Other genes**
Patient1	E3/E3	H1/H2	C C	GBA, PRKN, PINK1 negative
Patient2	E2/E3	H1/H1	T T	GBA, PRKN, PINK1 negative

Table S2: Genetic information of the two LRRK2 G2019S-PD patient donors.
Genetic characteristics of the two LRRK2 G2019S-PD patients for Alzheimer APOE haplotype, and haplotype of frequent single nucleotide polymorphisms in PD risk factors MAPT and SNCA.

Pat ID	Gender	Birth year	Age at onset	Age at examination	Disease duration	LEDD	Hoehn & Yahr Stadium	UPDRS_III Score	MoCA-Total value	CSF Abeta42 pg/ml	CSF h-Tau pg/ml	CSF p-Tau 181 pg/ml	CSF NfL pg/ml	CSF alpha synuclein total pg/ml	Number of positive seedings in 30h	RT-QuIC seeding result
Patient1	F	1931	70	75	5		2.5									
Patient1	F	1931	70	76	6		3									
Patient1	F	1931	70	77	7	120	2.5	41		547	421	53	1935			
Patient1	F	1931	70	78	8	380	3	60								
Patient1	F	1931	70	80	10	580	4	44	19	404	542	67		575.4	4	positive
Patient1	F	1931	70	80	10	760	3	56	24	450	556	81				
Patient1	F	1931	70	81	11		3									
Patient1	F	1931	70	83	13	675	3	63								
Patient1	F	1931	70	83	13		3		12							
Patient2	F	1958	40	50	10	940	2	13		1003	144	31				
Patient2	F	1958	40	52	12	1148		25	28	819	148	26				
Patient2	F	1958	40	53	13	1148	2	21	27	923	237	35	794	444.25	3	positive
Patient2	F	1958	40	54	14	894	2	24	26	794	200	32	830	267.15	4	positive
Patient2	F	1958	40	54	14		2		26							
Patient2	F	1958	40	55	15	1290	2	11	26							
Patient2	F	1958	40	64	24	1237	2	11	28							

Table S3: Demographic, clinical, and CSF biomarker information of the two LRRK2 G2019S-PD patient donors.
Characteristics of the two LRRK2 G2019S-PD patients for demographic, clinical, and CSF data. LEDD = Levodopa Equivalent Dose, UPDRS = Unified Parkinson`s Disease Rating Scale, MoCA = Montreal Cognitive Assessment, NfL = neurofilament, RT-QuIC = real-time quaking-induced conversion.
